# The effectiveness of digital physical activity interventions in older adults: a systematic umbrella review and meta-meta-analysis

**DOI:** 10.1186/s12966-024-01694-4

**Published:** 2024-12-18

**Authors:** Stephanie J. Alley, Kim M. Waters, Felix Parker, D. L. I. H. K. Peiris, Samantha Fien, Amanda L. Rebar, Corneel Vandelanotte

**Affiliations:** 1 Appleton Institute, School of Health, Medical and Applied Sciences, Central Queensland University, Building 7, Bruce Highway, Rockhampton, QLD 4701 Australia; 2 Appleton Institute, School of Health Medical and Applied Science, Central Queensland University, 151-171 Boundary Road, Ooralea, QLD 4740 Australia; 3 Department of Sport Science and Physical Education, University of Kelaniya, Kandy Road, Dalugama, Kelaniya, 11600 Sri Lanka; 4 School of Health Medical and Applied Science, Central Queensland University, 90-92 Sydney Street, Mackay, QLD 4740 Australia; 5 Research Cluster for Resilience and Wellbeing, Appleton Institute, Wayville, South Australia 5034 Australia; 6 Arnold School of Public Health, University of South Carolina, Columbia, SC 29208 USA

**Keywords:** Digital, Technology, Interventions, Apps, Activity trackers, Older adults, Aging, Physical activity, Steps, Health promotion

## Abstract

**Background:**

Physical activity is important for healthy ageing, however most older adults are inactive. Numerous reviews with a range of inclusion criteria have been conducted on digital interventions to promote physical activity in older adults, and a synthesis of these is needed. Therefore, the objective of this study is to conduct an umbrella review and meta-meta-analysis on the effectiveness of digital interventions to promote physical activity in older adults.

**Methods:**

Nine databases were searched from January 2010 to December 2023. Systematic reviews and meta-analyses of primary studies using digital physical activity interventions to target healthy older adults or clinical populations of older adults with a self-reported or device measured physical activity outcome were eligible for inclusion.

**Results:**

In total, 22 systematic reviews and meta-analyses covering 185 primary research papers were eligible for inclusion. The total number of participants across all primary studies was 28,198. Most (21, 95%) reviews and meta-analyses were rated as having a low or critically low AMSTAR-2 confidence rating. Of the 22 included systematic reviews, 13 (59%) conducted a meta-analysis and 10 (45%) conducted a narrative synthesis. Most systematic reviews with a narrative synthesis found strong evidence for a positive effect or moderate evidence for a positive effect for physical activity outcomes (7/9, 78%) and steps (3/3, 100%). The meta-meta-analysis of primary papers included in meta-analyses demonstrated a significant moderate effect for steps and a significant small effect for total PA and MVPA. The strength of effect did not vary by intervention components (activity tracker, app-based, SMS/phone, web-based, and face-to-face), population (primary or secondary prevention), control group (none, other digital intervention, or non-digital intervention), or outcome measurement (self-reported or device measured). Only 3 (14%) reviews included longer term follow up outcomes after the end of the intervention, with mixed results.

**Conclusions:**

Evidence from 22 reviews and meta-analyses suggests that digital physical activity interventions are effective at increasing physical activity in older adults. Further primary research is needed in adults 65 years and over exclusively, and with longer-term follow up of physical activity outcomes. Future reviews should include a published protocol and interpret results according to risk-of-bias.

**Supplementary Information:**

The online version contains supplementary material available at 10.1186/s12966-024-01694-4.

## Background

Physical activity is essential for healthy aging. Older adults who are physically active have a lower risk of chronic illness and falls, and have improved function, cognition, and mental health [1]. Despite this, most older adults are insufficiently active. Globally only 43.5% of older adults are meeting the recommendations to engage in 30 min of physical activity on most days [2]. The population is aging globally, placing an increasing burden on health care systems worldwide [3, 4]. Therefore, there is an urgent need for physical activity promotion in older adults to improve the health and wellbeing of older adults, and address the increasing burden on health care systems [4].

Physical activity interventions that are effective at increasing physical activity in large numbers of older adults are needed to address inactivity in this age group [5]. Traditional face-to-face physical activity interventions (e.g., counselling and group-based interventions) are effective at increasing physical activity in older adults; however, these interventions are costly due to the human resources required and they have geographical restrictions [6]. There has been a rise in digital physical activity interventions which have the potential to reach large numbers of people in multiple locations at a relatively low cost [7]. Digital interventions such as activity monitors, smartphone and tablet applications (apps), automated Short Message Service (SMS), chatbots, and computer-tailored advice are effective in the general population [8, 9] and show promise in older adults [10, 11]. Most older adults are now frequent internet users [12], and although the evidence is mixed, some studies suggest that older adults find digital physical activity interventions usable, acceptable, and engaging [11, 13–15]. Non usage attrition is however common in digital interventions and limits their effectiveness in the long-term [16]. Overall, the effectiveness of many digital physical activity interventions has been demonstrated in older adults; with mixed results for some digital interventions including long-term interventions and interventions that are entirely self-lead [17–19].

The literature on digital physical activity interventions is rapidly increasing, including in older adults [5, 20–24]. Multiple systematic reviews have investigated the effectiveness of digital interventions in older adults [5, 20–22]. Some focus on the effectiveness of specific types of digital interventions such as m-health (applications and SMS) [20] or wearable activity trackers [25]. Others include multiple types of digital interventions [21]. Inclusion criteria vary in disease status (healthy, chronic illness), comparison groups (usual care, face-to-face, no intervention) and intervention characteristics (multiple components, inclusion of face-to-face contact) [5].

A rapid review of reviews published in 2020 [5] found five reviews of digital physical activity interventions in older adults. They included reviews on the effectiveness of apps, websites, and activity monitors for promoting physical activity in older adults and found that there is low to moderate evidence that mHealth or eHealth physical activity interventions may be effective in older adults in the short term [5]. However, people with chronic conditions were excluded, additional reviews have been conducted since then, and no meta-meta-analysis was conducted. It is important to consider older adults with chronic diseases when determining the evidence for digital physical activity interventions in the older adult population. Many older adults live with a chronic illness or comorbidities [26], and physical activity not only reduces the risk of further chronic illness but can improve symptoms and outcomes of existing chronic illness [1]. Therefore, an updated overview of the literature is needed to synthesise findings of digital physical activity interventions in older adults with the inclusion of recent reviews, older adults with a chronic illness, and a meta-meta-analysis to present an overall estimate of effect. The current study aims to conduct an umbrella review of systematic reviews and a meta-meta-analysis to determine current available evidence on the effectiveness of digital physical activity interventions in older adults.

## Methods

### Search strategy

The PRISMA guidelines were followed in the conduct and reporting of this systematic umbrella review and meta-meta-analyses [27]. The databases searched included CINAHL Complete (via EBSCOhost), SPORTDiscus (via EBSCOhost), Web of Science, MEDLINE (via OVID), PubMed, PsycINFO (via OVID), Embase, Scopus, and Cochrane Library. The search strategy included both free text and MESH search terms around ‘older adults,’ ‘physical activity,’ ‘systematic review,’ and ‘digital interventions’ (incl., activity monitors, exergame, e-health, m-health, app-based, web-based) with Boolean logic used to combine the search terms (see Additional file 1 for a detailed search strategy for each database). Searches were conducted on 04/12/2023 and were limited to those published during or after 2010 as reviews published prior to 2010 predominantly include interventions tested using older digital technology before the release of the smartphone, modern websites with 2.0 features and advanced activity trackers. Searches were also limited to those written in the English language only. Reviews were only included where full text articles could be obtained. Forward and backward searches were conducted by manually screening citing papers and the reference lists of all included systematic reviews for further relevant reviews.

### Inclusion criteria

#### Types of study

Systematic reviews and meta-analyses of randomised controlled trials (RCTs), non-randomised controlled trials, and pre-post designs were included. Systematic reviews of cross sectional, cohort or any other non-experimental research designs were excluded. Reviews which were not systematically conducted (i.e., based on a clearly formulated question, identifies relevant studies, appraises quality and summarises the evidence by use of explicit methodology) were excluded. Review of reviews, meta-meta-analyses, umbrella reviews, scoping reviews, bibliometric analyses or other meta-review studies were excluded.

#### Participants/population

The target population were older adults, for which two criteria were applied. The reviews must either have an overall mean age of 60 years or over, or each of the individual studies included in the review must have a mean age of 50 years or over. These two criteria were applied as the reviews presented the information in different ways (e.g., review mean average or range of individual study mean ages). The cut points were chosen as many reviews on digital physical activity interventions in older adults purposefully have a low age cut point to avoid excluding studies that focus on aging adults. Only reviews on community dwelling older adults (i.e. not living in an aged care home) were included.

#### Intervention(s), exposure(s)

This systematic review focused on reviews of population based physical activity interventions that use digital technology to deliver intervention content to participants with limited involvement of program staff. Therefore, reviews on any intervention that uses digital technology to deliver all or most of the content to participants were included. This included but was not limited to activity trackers (e.g., Fitbits), websites, mobile apps, SMS, and exergame (incl., virtual reality, augmented reality).

#### Comparator(s)/control

Reviews including both inactive no intervention controls (incl., waitlist or usual care) and/or active controls receiving another physical activity intervention were included (incl., non-digital or other digital intervention). Pre-post designs without a control group were also included.

#### Main outcome(s)

Minutes of physical activity (incl., total physical activity, light intensity, moderate intensity, vigorous intensity, or moderate to vigorous intensity), frequency of physical activity (incl., frequency of cardio, strength, balance or flexibility sessions), percentage meeting physical activity guidelines, and number of steps were included as outcomes. Both self-report survey data and device (i.e. accelerometer) measured physical activity were included.

### Study selection

**Fig. 1:**
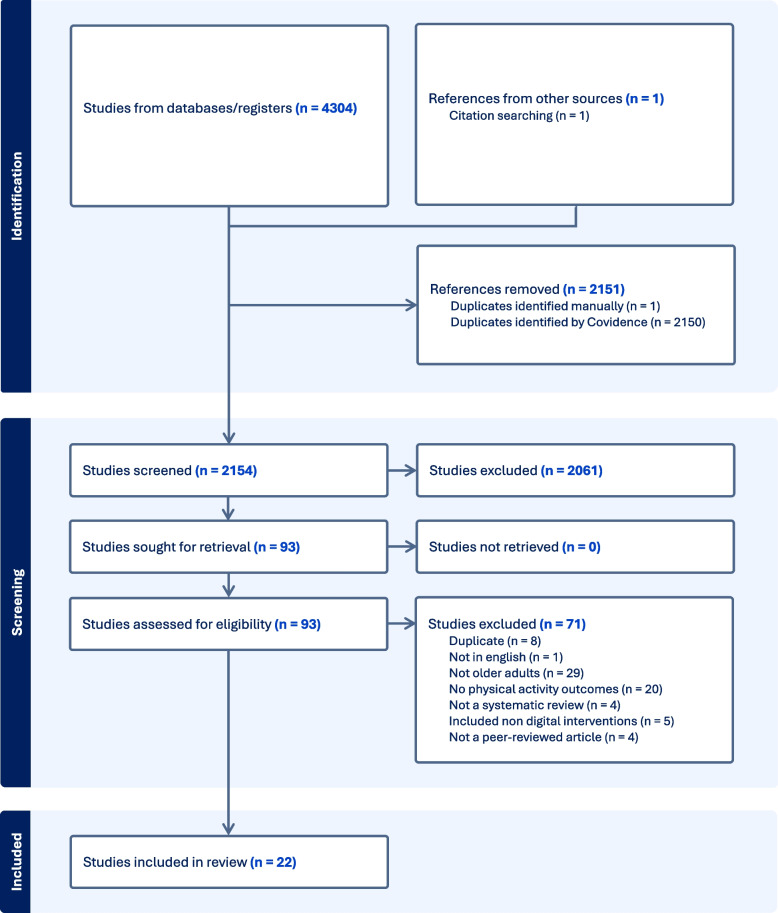
PRISMA flow diagram

Covidence online software (https://www.covidence.org/) was used for data management. Titles and abstracts of citations identified through database searches were screened for relevance by two independent reviewers and any disagreements were resolved by a third reviewer. The full text of the remaining reviews were screened by two independent reviewers and any disagreements were resolved by a third reviewer. Reasons for exclusions were recorded (see Additional file 2). Search results and screening are presented in Figure 1 (PRISMA flow diagram) [27].

### Data extraction

Data extraction of each included systematic review was conducted using a data collection form constructed in Covidence (see Additional file 3). Extraction was conducted by two independent researchers and any disagreements were resolved by discussion until a consensus was reached. Data extraction included publication details (author, year), review type (systematic review or meta-analysis), number of included studies, number of included participants, study designs (RCT, quasi-experimental, pre-post) participant characteristics (age, chronic disease), digital intervention components (activity trackers, websites, mobile apps, SMS), comparisons (no intervention, other digital intervention, non-digital intervention), and outcomes (self-reported or device measured minutes of physical activity and/or steps). Reviews were further categorized into primary prevention or secondary prevention and e-health, m-health or wearable activity tracker only. For systematic reviews, overall results were categorized as strong evidence for a positive effect (> = 75% of studies are significant, favours intervention), moderate evidence for a positive effect (> 50% of studies are significant, favours intervention), weak evidence for a positive effect (significant, favours intervention > significant favours control), strong evidence for a negative effect (> = 75% studies are significant, favours control), moderate evidence for a negative effect (> 50% significant, favours control), or weak evidence for a negative effect (significant favours control > significant, favours intervention) in line with past research [28]. For the meta-analyses, the number of studies, total number of participants, Standardised Mean Difference (SMD) (95% CI), Mean Difference, (MD) (95% CI) and heterogeneity (I^2^) were extracted for each physical activity outcome. The SMD, MD, and CI’s for each physical activity outcome, intervention components (activity tracker, mobile apps, SMS/phone, website, face-to-face), outcome measure (self-reported, wearable activity tracker), population (primary prevention, secondary prevention), and control group (no intervention, other digital intervention, non-digital intervention) were also extracted for each primary study included in the meta-analyses.

### Quality assessment

Quality of the included systematic reviews was assessed using the Assessment of Multiple Systematic Reviews v2 (AMSTAR-2) checklist [29]. The quality assessment for each systematic review was conducted by two independent reviewers with any disagreements resolved by discussion until a consensus was reached.

### Data synthesis

Meta-meta-analyses with Restricted Maximum Likelihood were conducted using multilevel modelling with the R 4.4.1 package *metafor* for the primary studies included in eligible meta-analyses for the outcomes of steps, MVPA, and total physical activity [30, 31]. To account for overlap of primary studies, duplicates were removed so that each primary study was only included once. All MD outcomes were converted to SMD, using pooled SD, assuming equal degrees of freedom across groups. Random effects were fixed to account for nesting of effects (and variance estimates) within-study and within-review. A set of models were estimated with the overall effect without other predictors, and then a set of models were estimated to test for moderation effect of intervention components (activity tracker vs app-based vs SMS/phone vs web-based vs face-to-face), outcome (tracker vs survey), population (primary prevention vs secondary prevention), and control group (usual care/none vs non-digital intervention vs other digital intervention).

### Deviations from registered protocol

Some changes were made to the registered protocol on PROSPERO (CRD42022345669). The first change was to remove physical function outcomes. This was due to the large number of falls prevention reviews that included physical capacity outcomes. This decision was made to keep the focus of this review on interventions to promote physical activity, rather than facilitate the delivery of clinical exercises. An additional age criterion was added to also include reviews with an overall mean age of 60 years or over, even if some studies included in the review had a mean age under 50 years of age. This was partially due to many reviews only reporting overall mean age, and partially due to some reviews focusing on an older demographic but including one or two studies with a younger mean age (Fig. 1).

## Results

### Study selection

Searches revealed 4305 records (*n* = 4304 from databases and *n* = 1 from citation searching). After automated removal of duplicates, 2154 studies remained. After title and abstract screening, 93 articles were screened in a full text review. A final 22 reviews were deemed eligible and were included in this review. A list of articles excluded at the full text screening stage with reasons for exclusion can be found in Additional file 2.

### Overview of included systematic reviews

The 22 included systematic reviews cover 185 primary research papers, 57 (31%) of which were represented in more than one review (range = 2–9). See Additional file 5 for a list of primary studies included in the reviews. Review characteristics and results are reported in Additional file 4. The median number of primary studies included in the reviews was 10 and ranged from 3 to 44. The median number of participants included in the reviews was 1556 and ranged from 322 to 6671. The total number of participants across all primary studies was 28,198. Only four reviews reported the overall mean age which ranged from 60–62 years. The reviews were published between 2015 and 2023 and the primary research papers included in the reviews were published between 2000 and 2022.

Of the 22 included reviews, eight (36%) included only primary prevention studies [19, 23, 32–37], nine (41%) included both primary and secondary prevention studies [15, 20–22, 38, 39, 14, 40, 41] and five (23%) included secondary prevention studies only [42–46]. Of the secondary prevention reviews, two focused on cardiovascular disease [42, 44], one on coronary heart disease specifically [46], one on non-communicable diseases [43], and one on cardiometabolic diseases [45].

All but one review focused on technology-based interventions only (21/22, 95%). One review included all types of physical activity interventions and only their sub-analysis of computer delivered interventions and wearable activity trackers is included in this review [36]. Seven reviews (32%) included interventions with wearable activity trackers [22, 23, 34, 38, 40, 42, 45], four (18%) included e-health interventions [35–37, 39], five (23%) included m- health interventions [15, 20, 32, 41, 44] and six (27%) included both e- and m-health interventions [19, 21, 14, 33, 43, 46]. All reviews examining e- and/or m-health studies also included interventions that used wearable activity trackers in conjunction with e- and/or m-health tools. One review only included studies that looked at the effectiveness of activity trackers [14]. Two of the reviews on m-health interventions, specifically focused on app-based interventions only (i.e., no SMS or other m-health interventions) [32, 44].

Of the 22 included reviews, 12 (54%) included only RCT’s [14, 19, 22, 23, 34, 35, 38–40, 42, 45, 46], five (23%) included both RCT’s and quasi-experimental designs [32, 33, 37, 41, 44], and five (23%) included RCT’s, quasi-experimental and pre-post designs [15, 20, 21, 36, 43]. Twelve (54%) included studies with any type of control [15, 19–21, 32, 34, 36–38, 41, 43, 44], four (18%) included only no intervention controls [23, 42, 45, 46], four (18%) included control groups who received either no intervention or another type of intervention [22, 33, 35, 39], and two (9%) reviews included a specific control group [40, 14]. One of these included only studies which had control groups who received a face-to-face intervention [14], and the other included only studies which compared wearable activity trackers with feedback to wearable activity trackers with no feedback [40]. Just under a third included only device measured physical activity measures (7/22, 32%) [19, 22, 23, 32, 34, 42, 45], and just over two-thirds included both self-reported and device measured physical activity outcome measures (15/22, 68%) [15, 20, 21, 33, 35, 36, 14, 37–41, 43, 44, 46].

Of the 22 included systematic reviews, 13 (59%) conducted a meta-analysis [21–23, 32, 34, 35, 38, 39, 14, 40, 42, 44, 45] and 10 (45%) conducted a narrative synthesis [15, 19, 20, 33, 34, 36, 37, 41, 43, 46]. One of these reviews conducted both a narrative synthesis and a meta-analysis [34], and one conducted a meta-analysis but was not included in the meta-meta-analysis as it did not report effect sizes and variance of the primary studies that they used in the meta-analysis [21]. The results for the narrative syntheses and meta-analyses are presented in Additional file 4 and the results of the meta-meta-analysis are presented in Tables 1 and 2. All but one meta-analysis (11/12, 92%) included results from studies with a control group. The meta-analysis that included pre-post designs presented this separately and was not included in the meta-meta-analysis [21]. Some narrative syntheses included trials both with and without a control group (4/10, 40%) [15, 20, 36, 43]. One of these presented results from pre-post and controlled trials separately [20].

**Table 1:** Multilevel meta-meta-analyses estimates for steps, MVPA, and total PA

	Steps	MVPA	Total PA
Review variance, σ^2^	0.01	0.00	0.02
Study variance, σ^2^	0.24	0.12	0.07
Q(*df*)	Q(*81*) = 677.67^*^	Q(34) = 171.67^*^	Q(53) = 194.30^*^
Estimate, *SMD* (95% CI)	0.52^*^ (0.39 – 0.65)	0.42^*^ (0.27 – 0.57)	0.28^*^ (0.16 – 0.40)

*MVPA* moderate-vigorous physical activity, *total PA* total physical activity, *Q* Cochran’s Q, *df* degrees of freedom, *SMD* standardised mean difference, *CI* confidence interval

^*^p < .05

**Table 2:** Multilevel meta-meta-analyses estimates for steps, MVPA, and total PA

	Steps	MVPA	Total PA
Review variance, σ^2^	0.03	0.00	0.01
Study variance, σ^2^	0.23	0.10	0.07
Heterogeneity, Q(*df*)	Q(70) = 480.82^*^	Q(24) = 83.79^*^	Q(44) = 142.33^*^
Moderators, QM(df)	QM(10) = 8.44	QM(10) = 18.11	QM(10) = 11.31
Intercept (95% CI)	1.58 (-0.13 – 3.33)	0.31 (-0.94 – 1.55)	0.70^*^ (0.03 – 1.38)
Outcome – survey	--	0.04 (-0.91 – 0.99)	-0.41 (-1.18 – 0.36)
Outcome – tracker (95% CI)	-0.94 (-2.02 – 0.13)	0.59 (-0.35 – 1.53)	-0.17 (-0.91 – 0.56)
Intervention – activity tracker (95% CI)	0.44 (-0.33 – 1.21)	0.05 (-0.40 – 0.50)	0.01 (-0.25 – 0.27)
Intervention – app-based (95% CI)	0.10 (-0.67 – 0.87)	0.04 (-0.43 – 0.52)	0.14 (-0.50 – 0.77)
Intervention – SMS/phone (95% CI)	-0.05 (-0.39 – 0.28)	0.42 (-0.07 – 0.90)	0.07 (-0.25 – 0.40)
Intervention – web-based (95% CI)	-0.26 (-0.68 – 0.16)	-0.14 (-0.51 – 0.23)	-0.11 (-0.41 – 0.19)
Intervention – face-to-face (95% CI)	-0.06 (-0.35 – 0.22)	-0.34 (-0.71 – 0.03)	-0.25^*^ (-0.49—-0.01)
Population – primary prevention (95% CI)	0.04 (-0.25 – 0.32)	-0.08 (-0.45 – 0.28)	0.01 (-0.23 – 0.24)
Control – non-digital intervention (95% CI)	-0.45 (-1.82 – 0.91)	--	0.01 (-0.22 – 0.25)
Control – other digital (95% CI)	-0.97 (-2.55 – 0.61)	-0.56 (-1.18 – 0.06)	--
Control – usual care/none (95% CI)	-0.51 (-1.92 – 0.89)	-0.13 (-0.57 – 0.32)	--

*MVPA* moderate-vigorous physical activity, *total PA* total physical activity, *Q* Cochran’s Q, *df* degrees of freedom, *SMD* standardised mean difference, *CI* confidence interval

^*^p < .05

### Quality assessment results

The completed AMSTAR-2 checklist for each review is reported in Additional file 6. Of the 22 included reviews 15 (68%) were categorized as a critically low confidence rating [15, 19, 20, 23, 32–34, 36, 38, 39, 41–44, 46], six (27%) were categorized as having a low confidence rating [21, 22, 35, 37, 14, 45] and one (5%) was categorized as having a high confidence rating [40]. The low ratings were mostly due to the reviews not including a list of excluded studies with reasons why (*n* = 17, 77%) [15, 19–23, 32–34, 36, 38, 39, 41–45], and not accounting for risk of bias when discussing/interpreting review results (*n* = 13, 59%) [15, 19, 20, 23, 33, 35–38, 41–44]. Further, many reviews did not have a published protocol (*n* = 7, 32%) [15, 19, 20, 38, 39, 41, 46]. The systematic reviews with a narrative synthesis had a higher percentage with a critically low confidence rating (*n* = 8, 89%) compared to the meta-analyses (*n* = 6, 50%). Due to most reviews having a low or critically low confidence rating, results could not be compared by quality rating.

### Results of narrative syntheses

Of the nine systematic reviews which presented a narrative synthesis of physical activity outcomes, five (56%) found strong evidence for a positive effect [15, 33, 34, 41, 43]. Moderate evidence for a positive effect for physical activity was found in two reviews (22%) [20, 36]. One of these broke their results down further and found weak evidence for a positive effect for secondary prevention and strong evidence for a positive effect for primary prevention [20]. Lastly, two reviews (22%) found weak evidence for a positive effect for physical activity [37, 46]. One of these examined e- and m-health for secondary prevention [46] and the other examined e-health for primary prevention [37]. These narrative synthesis results for physical activity did not vary by population, intervention, measurement, or comparison. Three reviews reported step results, of which one (33%) found strong evidence for a positive effect [36] and two (66%) found moderate evidence for a positive effect [19, 41]. The narrative synthesis results for steps did not vary by population, intervention, or comparison. One review which looked at e- and m-health for primary prevention broke findings down by physical activity intensity and found weak evidence for a positive effect for light PA, strong evidence for a positive effect for moderate PA, weak evidence for a negative effect for MVPA, and weak evidence for a positive effect for vigorous PA [19].

### Results of meta-meta-analyses

Of the 13 reviews that conducted a meta-analysis, 12 presented step outcomes [14, 21–23, 32, 34, 35, 39, 40, 42, 44, 45], six presented MVPA outcomes [21, 23, 34, 35, 40, 14], and five presented total PA outcomes [21, 23, 35, 38, 14]. Ten (83%) saw a significant improvement in steps [14, 22, 23, 35, 39, 40, 42, 44, 45, 47]. These reviews were a range of primary prevention, secondary prevention, wearable activity trackers, and e- and m-health interventions. The first review that did not see an improvement in steps examined six studies with *n* = 486 participants that used m-health (applications) for the primary prevention of physical activity [32]. The second review that did not see an improvement in steps examined 22 studies with *n* = 1757 participants that used e- or m-health interventions for either primary or secondary prevention of physical activity [21]. This review presented findings from RCT’s and pre-post designs separately and neither found significant improvements in steps [21]. Two reviews also presented step outcomes at follow up. One reviewed m-health interventions (specifically smartphone and tablet applications) and found no effectiveness at 6–12 month follow up (*n* = 2 studies) [32]. The other was a review of wearable activity trackers which found effectiveness at six months (*n* = 3 studies), but not at three months (*n* = 4 studies) follow up [22]. This review also broke down step results by primary and secondary prevention and found positive results for both but a larger effect for primary prevention [22].

Of the six meta-analyses that had MVPA as an outcome, four (67%) saw a significant improvement in MVPA [21, 23, 35, 40]. These included two primary prevention reviews [23, 35] and two that examined both primary and secondary prevention [21, 40]. Two examined wearable activity trackers [23, 40], one examined both e- and m-health [21], and one examined e-health [35]. One of these reviews [35] presented findings by mins per day and mins per week separately, and both were improved by the e-health intervention. The first meta-analysis that didn’t see a significant improvement in MVPA looked at three studies with *n* = 475 participants that used an e- or m-health intervention for primary or secondary prevention [14]. The second meta-analysis that didn’t see a significant improvement in MVPA looked at three studies with *n* = 201 participants that used a wearable activity tracker for primary prevention.

Of the five meta-analysis that had total PA as an outcome, five (100%) saw a significant improvement in total PA [14, 21, 23, 35, 38]. These included two primary prevention reviews [23, 35], and three reviews examining both primary and secondary prevention [21, 38, 14]. Two examined the effectiveness of wearable activity trackers [23, 38], two examined the effectiveness of e- or m-health interventions [21, 14] and one examined the effectiveness of e-health interventions [35]. One review presented findings from RCT’s and pre-post designs separately and found significant improvements in total PA in both designs [21]. One review presented results by accelerometers and pedometers with only accelerometers found to be effective at increasing total PA [38]. One review additionally presented follow up outcomes for Total PA [21]. The review demonstrated no follow up effects for e- and m-health interventions at 6–12 months (*n* = 2 studies).

The results of the meta-meta-analyses are shown in Tables 1 and 2. Forest plots of primary study effect sizes for steps, MVPA and total PA are presented in Additional file 7. Across all models, most variability was at the within-study level (Additional file 8 presents variance decomposition graph for each outcome). The meta-meta-analysis showed that all effects were statistically significant and had statistically significant variability (Table 1). The effect for steps was moderate, and the effect for total PA and MVPA were small (although they were not statistically significantly different from one another). Table 2 shows the results for the moderation analyses. The only significant moderation effect was that for total PA, digital interventions with a face-to-face component had a slightly smaller effect than those that did not include a face-to-face component. Note, however, that this moderation effect is small and the overall variability accounted by moderation effects in the model was not statistically significantly different from zero; therefore further research is needed to confirm this moderation effect and conclusions should not be generalised beyond our findings.

## Discussion

This systematic umbrella review and meta-meta-analysis aimed to give an overview of reviews looking at the effectiveness of digital physical activity interventions to promote physical activity in older adults. Overall, most reviews demonstrated the effectiveness of digital physical activity interventions for older adults including those using wearable activity trackers. The meta-meta-analysis found a significant moderate effect of digital physical activity interventions for increasing steps, and a significant small effect for increasing MVPA and total PA. Results did not differ by measurement type (self-reported or activity tracker). Most systematic reviews which conducted a narrative synthesis found strong or moderate evidence for a positive effect for physical activity outcomes (7/9, 78%) and steps (3/3, 100%). These results demonstrate that digital tools including e- and m-health, and activity trackers are effective for promoting physical activity in older adults. This finding is in line with a review of reviews of digital physical activity interventions for adults of all ages [24, 28] and a rapid review of reviews of e- and m-health for physical activity promotion in older adults conducted by McGarrigle and Todd [5]. McGarrigle and Todd [5] found four out of five reviews to support the effectiveness of e- and m-health for physical activity promotion in older adults. Our review extends on these findings to include 22 reviews including five with a focus on secondary prevention and 12 reviews published more recently.

The variance in effect sizes across meta-analyses was minimal, however, variability in effect sizes across studies was observed. Significant heterogeneity remained after accounting for intervention components, outcome measures, control group, and population. This indicates that there may be other factors unaccounted for that influence the size of effect of digital interventions. This may include the combination of components, behaviour change techniques, theoretical base, baseline levels of physical activity, the number of contacts with participants, and the length of the intervention. The effect of these factors observed in previous meta-analyses of physical activity interventions in adults of all ages is mixed [25, 48–51]. Therefore, it’s possible that such factors may influence the effectiveness of digital physical activity intervention in older adults, however future meta-analyses of primary studies with a range of specific moderators are required to determine this.

Few reviews included long-term follow up results (3/22, 14%), and those that did only included follow up results of a few primary studies. This is due to the lack of primary research in older adults looking at follow up outcomes of digital physical activity interventions. These review results demonstrated mixed evidence for long term effectiveness, therefore more primary research with outcomes assessed for maintenance of physical activity are needed. There is stronger evidence for the effectiveness of digital physical activity interventions at follow up in adults of all ages. A recent meta-meta-review concluded that e-and m-health interventions were effective at improving physical activity in adults of all ages at follow up, based on four meta-analyses and 47 included RCT’s that reported longer-term outcomes [24].

Many reviews included studies with mean ages as low as 50 years. Therefore, some participants would have been middle aged. However, as this was the lowest mean age allowed, the overall mean of participants included in each review was higher and included the target aging demographic in general. Ideally the age cut point for all participants would be 65 years of age minimum but due to a scarcity of research into digital physical activity interventions in this age group [17], no reviews had a cut point this high. This means that it is not clear if the overall results would differ if the focus was on adults 65 + years only. Few individual studies exist on digital physical activity interventions in adults exclusively 65 + years, and those that do also indicate the effectiveness of e- and m-health such as trackers and computer-tailored feedback in this age group [10, 17].

Of the 22 included reviews, only five focused on secondary prevention with the remainder including both primary and secondary prevention and primary prevention only. Both the synthesis of narrative reviews and meta-meta-analysis of primary papers revealed that effectiveness did not differ by the target population (i.e., primary or secondary prevention). One systematic review which conducted a narrative synthesis [20] did however conduct their analyses for primary and secondary prevention studies separately and found m-health interventions to be effective for physical activity promotion, but improved results for primary prevention (strong evidence for a positive effect) compared with secondary prevention (weak evidence for a positive effect). Despite this, previous research has consistently found digital tools to be effective at promoting physical activity in people with a range of diagnoses including heart disease, diabetes, osteoarthritis, chronic obstructive pulmonary disease populations for rehabilitation or general secondary prevention in participants of all ages [52].

Of the 22 included reviews, seven reviews (32%) included interventions with wearable activity trackers only, four (18%) included e-health interventions only, five (23%) included m-health interventions only and six (27%) included both e- and m-health interventions. The results of the reviews with a narrative synthesis did not appear to differ by the type of digital interventions included. In line with this, the meta-meta-analysis of primary studies included in the meta-analyses demonstrated that the size of effect did not vary by intervention components (activity tracker, app-based, SMS/phone, web-based, face-to-face). This is in line with findings from a recent meta-meta-analysis of e-and m-health physical activity interventions in adults of all ages and likely due to the overlap in intervention types [24]. Many e- and m-health interventions included an activity tracker as part of the intervention, and conversely many activity tracker-based interventions also included feedback or instruction through face-to-face or e- and/or m-health tools.

Around half of reviews included RCT’s only, with the other half also including pre-post designs and/or quasi-experimental designs. Most primary studies included in the reviews had controls with no intervention or usual care. Whilst most primary studies investigated the effectiveness of digital physical activity interventions for older adults compared to no intervention or usual care, the meta-meta-analysis demonstrated that effects did not differ between no intervention/usual care, other digital intervention and non-digital intervention controls (including group classes and face-to-face support). Previous research comparing the effectiveness of face-to-face compared to digital physical activity interventions in adults of all ages have mixed results [6, 53]. However, it is important to note that digital physical activity interventions have the benefit of fewer geographical restrictions and have the potential to be scaled-up at minimal cost per additional user [5, 24]. Whilst evidence from ecological trials and cost-effectiveness outcomes are lacking in adults of all ages [7, 54], the effectiveness demonstrated in this review indicates that they are a promising tool to promote physical activity in older adults. Future ecological trials and evaluation of cost-effectiveness outcomes are needed to determine the cost-effectiveness and scalability of digital physical activity interventions in older adults in real-world settings.

The quality ratings of all reviews were considered low according to the AMSTAR-2 ratings. This is similar to previous meta-reviews within the digital health field [28]. The low scores are due to potential biases introduced by not meeting critical elements, most commonly a lack of a published protocol, not including a list of excluded studies with reasons, or considering risk of bias when interpreting findings. It should be noted that including a list of excluded studies with reasons is not included in the PRISMA guidelines and could be considered to have a minimal influence on the quality of the review [27]. However, even if this was removed from the assessment, many studies still did not meet any other critical elements and would therefore still be categorized as having a critically low or low rating. The narrative that this score reflects quality of the reviews and is based on what the authors reported rather than what was actually done, the quality of included primary papers, and overall strength of evidence. Therefore, the AMSTAR-2 rating does not reflect overall strength of findings. One review had over 6000 participants, demonstrating high power of their findings, and other reviews included studies only with rigorous designs such as RCT’s with device measured physical activity outcomes. Nonetheless, future reviews in digital physical activity interventions should ensure they include a published protocol and consider risk of bias when interpreting findings.

The current study followed PRISMA guidelines for the conduct and reporting of the umbrella review and meta-meta-analysis and contributes to the literature by synthesising evidence from all available reviews on the effectiveness of digital physical activity interventions for older adults both with and without chronic disease. However, the low quality identified in most of the included reviews limits the trustworthiness of the evidence.

## Conclusions

The evidence from systematic reviews and meta-analyses of digital interventions to promote physical activity in older adults demonstrates their effectiveness at increasing steps, total PA, and MVPA in the short term. The meta-meta-analysis demonstrated that effectiveness did not differ by target population (secondary or primary prevention), intervention components (activity tracker, app-based, SMS/phone, web-based, face-to-face), control group (no intervention, other digital intervention or non-digital intervention), or physical activity measurement (self-report, activity tracker). Further primary research on digital physical activity interventions is needed in adults 65 + years exclusively, in an ecological setting, with cost-effectiveness outcomes and with long-term follow-up outcomes. Future reviews of digital physical activity interventions in older adults should include detailed moderators, a published protocol and consider risk of bias in the interpretation of results.

## Supplementary Information

Additional file 1. Search strategies for each database.

Additional file 2. List of excluded articles with reasons.

Additional file 3. Data extraction form.

Additional file 4. Table of review characteristics and results.

Additional file 5. List of studies included in reviews and number of reviews they were included in.

Additional file 6. AMSTAR-2 quality rating of included reviews.

Additional file 7. Forest plots of meta-meta-analysis results for total PA, steps and MVPA.

Additional file 8. Variance decomposition plots of meta-meta-analyses for steps, MVPA and total PA.

## Data Availability

Data sharing is not applicable to this article as no datasets were generated or analysed during the current study.

## Abbreviations

Apps: Smartphone and tablet applications
SMS: Short Message Service
MVPA: Moderate to Vigorous Physical Activity
Total PA: Total Physical Activity
RCT: Randomised Controlled Trial
CI: Confidence Interval
SMD: Standardised Mean Difference
