# Supplementary material for: The effectiveness of digital physical activity interventions in older adults: a systematic umbrella review and meta-meta-analysis
Source: Int J Behav Nutr Phys Act. 2024 Dec 18;21:144. doi: 10.1186/s12966-024-01694-4 (PMC11658456; doi:10.1186/s12966-024-01694-4)
Supplement: Supplementary file 1 — Additional file 1. Search strategies for each database. [file 12966_2024_1694_MOESM1_ESM.pdf]

Additional file 1: Search strategies for each database

Web of Science

1. (((((TS=(aged)) OR TS=(senior)) OR TS=(elder*)) OR TS=("older adult")) OR TS=("older adults")) OR TS=("older people")
2. ((((((((((((((((((((((((((((TS=(ehealth)) OR TS=(e-health)) OR TS=(mhealth)) OR TS=(m-health)) OR TS=("activity monitor")) OR TS=("activity monitors")) OR TS=("fitness tracker")) OR TS=("fitness trackers")) OR TS=("app based")) OR TS=("web based")) OR TS=(digital)) OR TS=(Wii)) OR TS=("virtual reality")) OR TS=("augmented reality")) OR TS=("mixed reality")) OR TS=("social networking")) OR TS=(Internet)) OR TS=(technolog*)) OR TS=("mobile health")) OR TS=("electronic health")) OR TS=("mobile app")) OR TS=("mobile application")) OR TS=(smartphone)) OR TS=(exergam*)) OR TS=(telehealth)) OR TS=(telemedicine)) OR TS=(wearable*)) OR TS=(tablet)) OR TS=(device)) OR TS=(computer)
3. ((((((((((((((((((((TS=("physical activity")) OR TS=(sport)) OR TS=(step*)) OR TS=(balance)) OR TS=(strength)) OR TS=(mobility)) OR TS=(aerobic)) OR TS=("resistance training")) OR TS=("resistance exercise")) OR TS=(gym*)) OR TS=(workout)) OR TS=(walk*)) OR TS=(fall*)) OR TS=("health behaviour")) OR TS=("health behavior")) OR TS=("healthy ageing")) OR TS=("active ageing")) OR TS=(gait)) OR TS=("health promotion")) OR TS=(fitness)) OR TS=("physical function")
4. (TS=("Systematic review")) OR TS=("meta analysis")
5. 1 and 2 and 3 and 4
6. Timespan: 2010-01-01 to 2023-12-04
7. English (Languages)

Scopus

((TITLE-ABS-KEY("Meta analysis")) OR (TITLE-ABS-KEY("Systematic review"))) AND (TITLE-ABS-KEY(("physical activity") OR (sport) OR (step*) OR (balance) OR (strength) OR (mobility) OR (aerobic) OR ("resistance training") OR ("resistance exercise") OR (walk*) OR (fall*) OR ("active ag?ing") OR (gait) OR ("fitness") OR ("physical function"))) AND (TITLE-ABS-KEY((ehealth) OR (e-health) OR (mhealth) OR (m-health) OR ("activity monitor") OR ("activity monitors") OR ("fitness tracker") OR ("fitness trackers") OR ("app based") OR ("web based") OR (digital) OR (Wii) OR ("virtual reality") OR ("augmented reality") OR ("mixed reality") OR ("social networking") OR (Internet) OR (technolog*) OR ("mobile health") OR ("electronic health") OR ("mobile app") OR ("mobile application") OR (smartphone) OR (exergam*) OR (telehealth) OR (wearable*) OR (tablet) OR (device) OR (computer))) AND (TITLE-ABS-KEY(senior OR elder* OR "older adult" OR "older adults" OR "older people")) AND PUBYEAR > 2010 AND PUBYEAR < 2023 AND (LIMIT-TO (LANGUAGE,"English"))

SportDiscus and CINHAL (EBSCOhost)

(((((MH "Aged+") OR (MH "Aged, 80 and Over+") OR aged OR senior OR elder* OR "older adult" OR "older adults" OR "older people") AND ((MH "Computer Systems+") OR (MH "Digital Technology+") OR (MM "Wireless Communications") OR (MM "Mobile Applications") OR (MM "Smartphone") OR (MM "Exergames") OR (MH "Telemedicine+") OR (MH "Text Messaging+") OR (MM "Fitness Trackers") OR ehealth OR e-health OR mhealth OR m-health OR "activity monitor" OR "activity monitors" OR "fitness tracker" OR "fitness trackers" OR "app based" OR "web based" OR digital OR wii OR "virtual reality" OR "augmented reality" OR "mixed reality" OR

"social networking" OR Internet OR technolog* OR "mobile health" OR "electronic health" OR "mobile app" OR "mobile application" OR smartphone OR exergam* OR telehealth OR Telemedicine OR wearable* OR tablet OR device OR computer)) AND ((MH "Exercise+") OR (MH "Gait+") OR (MH "Balance, Postural+") OR (MH "Physical Fitness+") OR (MM "Accidental Falls") OR (MH "Health Promotion+") OR (MH "Health Behavior+") OR "physical activity" OR sport OR step* OR balance OR strength OR mobility OR aerobic OR "resistance training" OR "resistance exercise" OR gym* OR workout OR walk* OR fall* OR "health behaviour" OR "health behavior" OR "healthy ageing" OR "active ageing" OR gait OR health promotion OR fitness OR "physical function")) AND (MM "Meta Analysis") OR (MM "Systematic Review") OR "systematic review" OR "meta analysis" OR PT "meta analysis" OR PT "systematic review"))

Limiters - published date: 20100101-; English Language

Search modes - Boolean/Phrase

Ovid and PsycINFO (MEDLINE)

1. exp Meta-Analysis as topic/ OR exp Systematic Reviews as Topic/ OR "systematic review".tw. OR "meta analysis".tw. OR "systematic review".pt. OR "meta analysis".pt.
2. exp Aged/ OR exp "Aged, 80 and over"/ OR exp "Aged, 80 and over"/ OR elder*.tw. OR "older adult".tw. OR "older adults".tw. OR "older people".tw.
3. exp Digital Technology/ OR exp Wireless Technology/ OR exp Mobile Applications/ OR exp Smartphone/ OR exp Exergaming/ OR exp Text Messaging/ OR exp Fitness Trackers/ OR ehealth.tw. OR e-health.tw. OR mhealth.tw. OR m-health.tw. OR "activity monitor".tw. OR "activity monitors".tw. OR "fitness tracker".tw. OR "fitness trackers".tw. OR "app based".tw. OR "web based".tw. OR digital.tw. OR Wii.tw. OR "virtual reality".tw. OR "augmented reality".tw. OR "mixed reality".tw. OR "social networking".tw. OR Internet.tw. OR technolog*.tw. OR "mobile health".tw. OR "electronic health".tw. OR "mobile app".tw. OR "mobile application".tw. OR smartphone.tw. OR exergam*.tw. OR telehealth.tw. OR wearable*.tw. OR tablet.tw. OR device.tw.
4. exp Exercise/ OR exp Gait/ OR exp Postural Balance/ OR exp Physical Fitness/ OR exp Accidental Falls/pc [Prevention & Control] OR "physical activity".tw. OR sport.tw. OR step*.tw. OR balance.tw. OR strength.tw. OR mobility.tw. OR aerobic.tw. OR "resistance training".tw. OR "resistance exercise".tw. OR walk*.tw. OR fall*.tw. OR "active ag?ing".tw. OR gait.tw. OR fitness.tw. OR "physical function".tw.
5. 1 and 2 and 3 and 4
6. Limit 5 to (english language and yr="2010 -Current")

Pubmed

((("digital technology"[MeSH Terms]) OR ("wireless technology"[MeSH Terms]) OR ("mobile applications"[MeSH Terms]) OR ("smartphone"[MeSH Terms]) OR ("exergaming"[MeSH Terms]) OR ("text messaging"[MeSH Terms]) OR ("fitness trackers"[MeSH Terms])) OR (ehealth[tw]) OR (e-health[tw]) OR (mhealth[tw]) OR (m-health[tw]) OR ("activity monitor"[tw]) OR ("activity monitors"[tw]) OR ("fitness tracker"[tw]) OR ("fitness trackers"[tw]) OR ("app based"[tw]) OR ("web based"[tw]) OR (digital[tw]) OR (Wii[tw]) OR ("virtual reality"[tw]) OR ("augmented reality"[tw]) OR ("mixed reality"[tw]) OR ("social networking"[tw]) OR (Internet[tw]) OR (technolog*[tw]) OR ("mobile health"[tw]) OR ("electronic health"[tw]) OR ("mobile app"[tw]) OR ("mobile application"[tw]) OR (smartphone[tw]) OR (exergam*[tw]) OR (telehealth[tw]) OR (wearable*[tw]) OR (tablet[tw]) OR (device[tw]))) AND ((("aged"[MeSH Terms]) OR ("aged, 80 and over"[MeSH Terms])) OR (senior[tw]) OR (elder[tw]) OR ("older adult"[tw]) OR ("older adults"[tw]) OR ("older people"[tw]))) AND

((("exercise"[MeSH Terms]) OR ("gait"[MeSH Terms]) OR ("postural balance"[MeSH Terms]) OR ("physical fitness"[MeSH Terms]) OR ("accidental falls/prevention and control"[MeSH Major Topic]) OR ("health promotion"[MeSH Terms]) OR (health behavior[MeSH Terms]) OR ("physical activity"[tw]) OR (sport[tw]) OR (step*[tw]) OR (balance[tw]) OR (strength[tw]) OR (mobility[tw]) OR (aerobic[tw]) OR ("resistance training"[tw]) OR ("resistance exercise"[tw]) OR (gymnasium[tw]) OR (walk*[tw]) OR (fall*[tw]) OR ("active ageing"[tw]) OR (gait[tw]) OR ("fitness"[tw]) OR ("physical function"[tw]))) AND ((("systematic reviews as topic"[MeSH Terms]) OR ("systematic review"[Publication Type]) OR ("meta analysis as topic"[MeSH Terms]) OR ("meta analysis"[Publication Type]) OR ("meta analysis"[All Fields]) OR ("systematic review"[All Fields]))) AND (((2010:3000/12/12[pdat]) AND (english[Filter])))

Cochrane

1. MeSH descriptor: [Aged] explode all trees OR MeSH descriptor: [Aged, 80 and over] explode all trees OR (aged):ti,ab,kw OR (senior):ti,ab,kw OR (elder*):ti,ab,kw OR ("older adult"):ti,ab,kw OR ("older adults"):ti,ab,kw OR ("older people"):ti,ab,kw
2. MeSH descriptor: [Computers] explode all trees OR MeSH descriptor: [Digital Technology] explode all trees OR MeSH descriptor: [Wireless Technology] explode all trees OR MeSH descriptor: [Mobile Applications] explode all trees OR MeSH descriptor: [Smartphone] explode all trees OR MeSH descriptor: [Exergaming] explode all trees OR MeSH descriptor: [Telemedicine] explode all trees OR MeSH descriptor: [Text Messaging] explode all trees OR MeSH descriptor: [Fitness Trackers] explode all trees OR (ehealth):ti,ab,kw OR (e-health):ti,ab,kw OR (mhealth):ti,ab,kw OR (m-health):ti,ab,kw OR ("activity monitors"):ti,ab,kw OR ("activity monitor"):ti,ab,kw OR ("fitness trackers"):ti,ab,kw OR ("fitness tracker"):ti,ab,kw OR ("app based"):ti,ab,kw OR ("web based"):ti,ab,kw OR (digital):ti,ab,kw OR (Wii):ti,ab,kw OR ("virtual reality"):ti,ab,kw OR ("augmented reality"):ti,ab,kw OR ("mixed reality"):ti,ab,kw OR ("social networking"):ti,ab,kw OR (Internet):ti,ab,kw OR (technolog*):ti,ab,kw OR ("mobile health"):ti,ab,kw OR ("electronic health"):ti,ab,kw OR ("mobile app"):ti,ab,kw OR ("mobile application"):ti,ab,kw OR (smartphone):ti,ab,kw OR (exergam*):ti,ab,kw OR (telehealth):ti,ab,kw OR (telemedicine):ti,ab,kw OR (wearable*):ti,ab,kw OR (tablet):ti,ab,kw OR (device):ti,ab,kw OR (computer):ti,ab,kw
3. MeSH descriptor: [Exercise] explode all trees OR MeSH descriptor: [Gait] explode all trees OR MeSH descriptor: [Postural Balance] explode all trees OR MeSH descriptor: [Physical Fitness] explode all trees OR MeSH descriptor: [Accidental Falls] explode all trees OR MeSH descriptor: [Health Promotion] explode all trees OR MeSH descriptor: [Health Behavior] explode all trees OR ("physical activity"):ti,ab,kw OR (sport):ti,ab,kw OR (step*):ti,ab,kw OR (balance):ti,ab,kw OR (strength):ti,ab,kw OR (mobility):ti,ab,kw OR (aerobic):ti,ab,kw OR ("resistance training"):ti,ab,kw OR ("resistance exercise"):ti,ab,kw OR (gym*):ti,ab,kw OR (workout):ti,ab,kw OR (walk*):ti,ab,kw OR (fall*):ti,ab,kw OR ("health behaviour"):ti,ab,kw OR ("health behavior"):ti,ab,kw OR ("healthy ageing"):ti,ab,kw OR ("active ageing"):ti,ab,kw OR (gait):ti,ab,kw OR ("health promotion"):ti,ab,kw OR (fitness):ti,ab,kw OR ("physical function"):ti,ab,kw
4. 1 and 2 and 3
5. (systematic review):pt OR ("meta-analysis"):pt OR ("systematic review"):ti,ab,kw OR ("meta analysis"):ti,ab,kw
6. 1 and 2 and 3 and 5
7. Limit 4 and 6 to Cochrane Library publication date Between Jan 2010 and Dec 2022, in Cochrane Reviews

Embase

1. ‘aged’/exp OR 'very elderly'/exp OR senior:ab,ti OR elder*:ab,ti OR 'older adult':ab,ti OR 'older adults':ab,ti OR 'older people':ab,ti
2. 'exercise'/exp OR 'gait'/exp OR 'body equilibrium'/exp OR 'fitness'/exp OR 'falling'/exp OR 'physical activity':ab,ti OR sport:ab,ti OR step*:ab,ti OR balance:ab,ti OR strength:ab,ti OR mobility:ab,ti OR aerobic:ab,ti OR 'resistance training':ab,ti OR 'resistance exercise':ab,ti OR walk*:ab,ti OR fall*:ab,ti OR 'active ag?ing':ab,ti OR gait:ab,ti OR fitness:ab,ti OR 'physical function':ab,ti
3. 'digital technology'/exp OR 'wireless communication'/exp OR 'mobile application'/exp OR 'smartphone'/exp OR 'exergaming'/exp OR 'text messaging'/exp OR 'activity tracker'/exp OR ehealth:ab,ti OR 'e-health':ab,ti OR 'm-health':ab,ti OR 'ehealth':ab,ti OR 'activity monitor':ab,ti OR 'activity monitors':ab,ti OR 'fitness tracker':ab,ti OR 'fitness trackers':ab,ti OR 'app based':ab,ti OR 'web based':ab,ti OR digital:ab,ti OR 'wii':ab,ti OR 'virtual reality':ab,ti OR 'augmented reality':ab,ti OR 'mixed reality':ab,ti OR 'social networking':ab,ti OR internet:ab,ti OR technolog*:ab,ti OR 'mobile health':ab,ti OR 'electronic health':ab,ti OR 'mobile app':ab,ti OR :ab,ti OR telehealth:ab,ti OR wearable*:ab,ti OR tablet:ab,ti OR device:ab,ti
4. 'systematic review (topic)'/exp OR 'meta analysis (topic)'/exp OR 'meta analysis':ab,ti OR 'systematic review':ab,ti
5. 1 and 2 and 3 and 4
6. Limit 5 to (2010:py OR 2011:py OR 2012:py OR 2013:py OR 2014:py OR 2015:py OR 2016:py OR 2017:py OR2018:py OR 2019:py OR 2020:py OR 2021:py OR 2022:py)
