# Supplementary material for: The effectiveness of digital physical activity interventions in older adults: a systematic umbrella review and meta-meta-analysis
Source: Int J Behav Nutr Phys Act. 2024 Dec 18;21:144. doi: 10.1186/s12966-024-01694-4 (PMC11658456; doi:10.1186/s12966-024-01694-4)
Supplement: Supplementary file 2 — Additional file 2. List of excluded articles with reasons. [file 12966_2024_1694_MOESM2_ESM.pdf]

Additional file 2: List of excluded articles with reasons

	Reference	Reason
1.	Aalbers, T., Baars, M. A. E., & Rikkert, M. G. M. O. (2011). Characteristics of effective internet-mediated interventions to change lifestyle in people aged 50 and older: A systematic review. *Ageing Research Reviews*, *10*(4), 487-497.	Not older adults
2.	Abbaspur-Behbahani, S., Monaghesh, E., Hajizadeh, A., & Fehresti, S. (2022). Application of mobile health to support the elderly during the COVID-19 outbreak: A systematic review. *Health Policy and Technology*, *11*(1), 100595-100595	Not a systematic review
3.	Alharbi, M., Straiton, N., Smith, S., Neubeck, L., & Gallagher, R. (2019). Data management and wearables in older adults: A systematic review. *Maturitas*, *124*, 100-110.	No physical activity outcomes
4.	Andre, L., Giulioli, C., Piau, A., Bongard, V., Richard, E., Moll van Charante, E. P., Coley, N., & Andrieu, S. (2022). Telephone and smartphone-based interventions for cognitive and cardio-metabolic health in middle-aged and older adults: A systematic review. *Clinical Interventions in Aging*, *17*, 1599-1624	Included non-digital interventions
5.	Beishuizen, C. R. L., Stephan, B. C. M., van Gool, W. A., Brayne, C., Peters, R. J. G., Andrieu, S., Kivipelto, M., Soininen, H., Busschers, W. B., Moll van Charante, E. P., & Richard, E. (2016). Web-Based interventions targeting cardiovascular risk factors in middle-aged and older people: A systematic review and meta-analysis. Journal of Medical Internet Research, 18(3), e55-e55.	Not older adults
6.	Beishuizen, C. R., van Gool, W. A., Busschers, W. B., Peters, R. J., Moll van Charante, E. P., & Richard, E. (2016). Web-based interventions targeting cardiovascular risk factors in older people; a systematic review and meta-analysis. *Dutch Journal of Medicine*, *160*, D581.	Not older adults
7.	Bentlage, E., Nyamadi, J. J., & Dubbeldam, R. (2023). The importance of activating factors in physical activity interventions for older adults using information and communication technologies: Systematic Review. *JMIR mHealth and uHealth*, *11*, e42968-e42968.	No physical activity outcomes
8.	Bevilacqua, R., Casaccia, S., Cortellessa, G., Astell, A., Lattanzio, F., Corsonello, A., D'Ascoli, P., Paolini, S., Di Rosa, M., Rossi, L., & Maranesi, E. (2020). Coaching through technology: A systematic review into efficacy and effectiveness for the ageing population. *International Journal of Environmental Research and Public Health*, *17*(16), 5930-	No physical activity outcomes
9.	Binarelli, G., Joly, F., Tron, L., Lefevre Arbogast, S., & Lange, M. (2021). Management of cancer-related cognitive impairment: A systematic review of computerized cognitive stimulation and computerized physical activity. *Cancers*, *13*(20), 5161-	No physical activity outcomes

10. Blount, D. S., McDonough, D. J., & Gao, Z. (2021). Effect of wearable technology-based physical activity interventions on breast cancer survivors' physiological, cognitive, and emotional outcomes: A systematic review. *Journal of Clinical Medicine*, *10*(9), 2015-	Not older adults
11. Bossen, D., Veenhof, C., Dekker, J., & de Bakker, D. (2014). The effectiveness of self-guided web-based physical activity interventions among patients with a chronic disease: a systematic review. *Journal of Physical Activity & Health*, *11*(3), 665-677	Not older adults
12. Changizi, M., & Kaveh, M. H. (2017). Effectiveness of the mHealth technology in improvement of healthy behaviors in an elderly population-a systematic review. *mHealth*, *3*, 51-51.	Not older adults
13. Chaudhry, U. A. R., Wahlich, C., Fortescue, R., Cook, D. G., Knightly, R., & Harris, T. (2020). The effects of step-count monitoring interventions on physical activity: systematic review and meta-analysis of community-based randomised controlled trials in adults. *The International Journal of Behavioral Nutrition and Physical Activity*, *17*(1), 129-129.	Not older adults
14. Corregidor-Sánchez, A. I., Segura-Fragoso, A., Rodríguez-Hernández, M., Criado-Alvarez, J. J., González-Gonzalez, J., & Polonio-López, B. (2020). Can exergames contribute to improving walking capacity in older adults? A systematic review and meta-analysis. *Maturitas*, *132*, 40-48.	No physical activity outcomes
15. Davergne, T., Pallot, A., Dechartres, A., Fautrel, B., & Gossec, L. (2019). Use of wearable activity trackers to improve physical activity behavior in patients with rheumatic and musculoskeletal diseases: A systematic review and meta-analysis. *Arthritis Care & Research (2010)*, *71*(6), 758-767.	Not older adults
16. de Bruin, E. D., Hartmann, A., Uebelhart, D., Murer, K., & Zijlstra, W. (2008). Wearable systems for monitoring mobility-related activities in older people: a systematic review. *Clinical Rehabilitation*, *22*(10–11), 878-895.	No physical activity outcomes
17. Ester, M., Eisele, M., Wurz, A., McDonough, M. H., McNeely, M., & Culos-Reed, S. N. (2021). Current evidence and directions for future research in eHealth physical activity interventions for adults affected by cancer: Systematic review. *JMIR Cancer*, *7*(3), e28852-e28852.	Not older adults
18. Feter, N., dos Santos, T. S., Caputo, E. L., & da Silva, M. C. (2019). What is the role of smartphones on physical activity promotion? A systematic review and meta-analysis. *International Journal of Public Health*, *64*(5), 679-690.	Not older adults
19. Franssen, W. M. A., Franssen, G. H. L. M., Spaas, J., Solmi, F., & Eijnde, B. O. (2020). Can consumer wearable activity tracker-based interventions improve physical activity and cardiometabolic health in patients with chronic diseases? A systematic review and meta-analysis of randomised controlled trials. *The International Journal of Behavioral Nutrition and Physical Activity*, *17*(1), 57-57.	Not older adults
20. Geraedts, H., Zijlstra, A., Bulstra, S. K., Stevens, M., & Zijlstra, W. (2013). Effects of remote feedback in home-based physical activity interventions for older adults: a systematic review. *Patient Education and Counseling*, *91*(1), 14-24.	Included non-digital interventions

21. Goode, A. D., Lawler, S. P., Brakenridge, C. L., Reeves, M. M., & Eakin, E. G. (2015). Telephone, print, and Web-based interventions for physical activity, diet, and weight control among cancer survivors: a systematic review. *Journal of Cancer Survivorship*, *9*(4), 660-682.	Not older adults
22. Hall, A. K., Chavarria, E., Maneeratana, V., Chaney, B. H., & Bernhardt, J. M. (2012). Health benefits of digital videogames for older adults: A systematic review of the literature. *Games for health journal*, *1*(6), 402-410.	No physical activity outcomes
23. Hodkinson, A., Kontopantelis, E., Adeniji, C., van Marwijk, H., McMillian, B., Bower, P., & Panagioti, M. (2021). Interventions using wearable physical activity trackers among adults with cardiometabolic conditions: A systematic review and meta-analysis. *JAMA Network Open*, *4*(7), e2116382-e2116382.	Not older adults
24. Hosseinpour, M., & Terlutter, R. (2019). Your personal motivator is with you: A systematic review of mobile phone applications aiming at increasing physical activity. *Sports Medicine (Auckland)*, *49*(9), 1425-1447.	Not older adults
25. Janhunen, M., Karner, V., Katajapuu, N., Niiranen, O., Immonen, J., Karvanen, J., Heinonen, A., & Aartolahti, E. (2021). Effectiveness of exergame intervention on walking in older adults: A systematic review and meta-analysis of randomized controlled trials. *Physical Therapy*, *101*(9), 1-.	No physical activity outcomes
26. Jin, D., Halvari, H., Maehle, N., & Olafsen, A. H. (2022). Self-tracking behaviour in physical activity: a systematic review of drivers and outcomes of fitness tracking. *Behaviour & Information Technology*, *41*(2), 242-261.	Not older adults
27. Jones, K., Justice, J., Bess, B., Smith, A., & Kunard, J. (2021). Does wearable technology impact lifestyle change and physical activity in at-risk populations? a systematic review. [Abstract from Cardiopulmonary Physical Therapy Journal], 32*(3),* e12-e32.	Not a peer-reviewed article
28. Kappen, D. L., Mirza-Babaei, P., & Nacke, L. E. (2019). Older adults' physical activity and exergames: A systematic review. *International Journal of Human-Computer Interaction*, *35*(2), 140-167.	No physical activity outcomes
29. Khoo, S., Mohbin, N., Ansari, P., Al-Kitani, M., & Müller, A. M. (2021). mHealth interventions to address physical activity and sedentary behavior in cancer survivors: A systematic review. *International Journal of Environmental Research and Public Health*, *18*(11), 5798-.	Not older adults
30. Kim, Y., Hong, S., & Choi, M. (2022). Effects of serious games on depression in older adults: Systematic review and meta-analysis of randomized controlled trials. *Journal of Medical Internet Research*, *24*(9), e37753-e37753.	No physical activity outcomes
31. Koivisto, J., & Malik, A. (2021). Gamification for older adults: A systematic literature review. *The Gerontologist*, *61*(7), e360-e372.	No physical activity outcomes
32. Kos, M., Pijnappel, E. N., Buffart, L. M., Balvers, B. R., Kampshoff, C. S., Wilmink, J. W., van Laarhoven, H. W. M., & van Oijen, M. G. H. (2021). The association between wearable activity	Not older adults

monitor metrics and performance status in oncology: a systematic review. *Supportive Care in Cancer*, *29*(11), 7085-7099.	
33. Kurt, I., Tanriverdi, M. (2018). Effect of Virtual reality equipment by active video gaming on physical activity. *Journal of Exercise Therapy & Rehabilitation,* Supp(2), S-15.	Not a peer-reviewed article
34. Larsen, R. T., Christensen, J., Juhl, C. B., Andersen, H. B., & Langberg, H. (2019). Physical activity monitors to enhance amount of physical activity in older adults - a systematic review and meta-analysis. *European Review of Aging and Physical Activity*, *16*(1), 7-7.	Not older adults
35. Larsen, L. H., Schou, L., Lund, H. H., & Langberg, H. (2013). The physical effect of exergames in healthy elderly-A systematic review. *Games for health journal*, *2*(4), 205-212.	No physical activity outcomes
36. Liu, J. Y.-W., Kor, P. P.-K., Chan, C. P.-Y., Kwan, R. Y.-C., & Cheung, D. S.-K. (2021). Corrigendum to 'The effectiveness of a Wearable Activity Tracker (WAT)-based Intervention to improve physical activity levels in sedentary older adults: A systematic review and meta-analysis' [Archives of Gerontology and Geriatrics, Volume 91, November–December 2020, 104211]. *Archives of Gerontology and Geriatrics*, *95*, 104420-104420.	Not a peer-reviewed article
37. Lynch, E. A., Jones, T. M., Simpson, D. B., Fini, N. A., Kuys, S. S., Borschmann, K., Kramer, S., Johnson, L., Callisaya, M. L., Mahendran, N., Janssen, H., & English, C. (2018). Activity monitors for increasing physical activity in adult stroke survivors. *Cochrane Database of Systematic Reviews*, *7*(7), CD012543–CD012543.	Not older adults
38. Mansi, S., Milosavljevic, S., Baxter, G. D., Tumilty, S., & Hendrick, P. (2014). A systematic review of studies using pedometers as an intervention for musculoskeletal diseases. *BMC Musculoskeletal Disorders*, *15*(1), 231-231.	Not older adults
39. Marin, T. S., Kourbelis, C., Foote, J., Newman, P., Brown, A., Daniel, M., Coffee, N. T., Nicholls, S. J., Ganesan, A., Versace, V. L., Beks, H., Haedtke, C. A., & Clark, R. A. (2019). Examining adherence to activity monitoring devices to improve physical activity in adults with cardiovascular disease: A systematic review. *European Journal of Preventive Cardiology*, *26*(4), 382-397.	Not older adults
40. Markert, C., Sasangohar, F., Mortazavi, B. J., & Fields, S. (2021). The use of telehealth technology to support health coaching for older adults: Literature review. *JMIR Human Factors*, *8*(1), e23796-e23796.	Included non-digital interventions
41. Michaelchuk, W., Oliveira, A., Marzolini, S., Nonoyama, M., Maybank, A., Goldstein, R., & Brooks, D. (2022). Design and delivery of home-based telehealth pulmonary rehabilitation programs in COPD: A systematic review and meta-analysis. *International Journal of Medical Informatics (Shannon, Ireland)*, *162*, 104754-104754.	No physical activity outcomes
42. Mönninghoff, A., Kramer, J. N., Hess, A. J., Ismailova, K., Teepe, G. W., Tudor Car, L., Müller-Riemenschneider, F., & Kowatsch, T. (2021). Long-term effectiveness of mHealth physical activity	Not older adults

interventions: Systematic review and meta-analysis of randomized controlled trials. *Journal of Medical Internet Research*, *23*(4), e26699-e26699.	
43. Muellmann, S., Forberger, S., Möllers, T., Bröring, E., Zeeb, H., & Pischke, C. (2016). EHealth interventions for the promotion of physical activity in older adults: A systematic review. *European Journal of Public Health*, *26*(suppl_1).	Not a systematic review
44. Müller, A. M., & Khoo, S. (2014). Non-face-to-face physical activity interventions in older adults: A systematic review. *The International Journal of Behavioral Nutrition and Physical Activity*, *11*(1), 35-35.	Included non-digital interventions
45. Ocagli, H., Agarinis, R., Azzolina, D., Todino, F., Binutti, M., Zabotti, A., Gregori, D., & Quartuccio, L. (2022). POS0163 Usefulness of wearable devices to assess physical activity in non-inflammatory and inflammatory rheumatic diseases: A systematic review and meta-analysis. *Annals of the Rheumatic Diseases*, *81*(Suppl 1), 310-311.	Not a peer-reviewed article
46. Ocagli, H., Agarinis, R., Azzolina, D., Zabotti, A., Treppo, E., Francavilla, A., Bartolotta, P., Todino, F., Binutti, M., Gregori, D., & Quartuccio, L. (2023). Physical activity assessment with wearable devices in rheumatic diseases: a systematic review and meta-analysis. *Rheumatology (Oxford, England)*, *62*(3), 1031-1046.	No physical activity outcomes
47. Portz, J. D., Miller, A., Foster, B., & Laudeman, L. (2016). Persuasive features in health information technology interventions for older adults with chronic diseases: a systematic review. *Health and Technology*, *6*(2), 89-99.	No physical activity outcomes
48. Rodriguez Rivas, A., & Rodriguez-Martin, B. (2020). Effectiveness of multicomponent interventions to promote physical activity in the elderly: A systematic review. *Gerokomos*, *31*(3), 149-157.	Not in English
49. Robbins, T. D., Lim Choi Keung, S. N., & Arvanitis, T. N. (2018). E-health for active ageing; A systematic review. *Maturitas*, *114*, 34-40.	No physical activity outcomes
50. Roberts, A. L., Fisher, A., Smith, L., Heinrich, M., & Potts, H. W. W. (2017). Digital health behaviour change interventions targeting physical activity and diet in cancer survivors: a systematic review and meta-analysis. *Journal of Cancer Survivorship*, *11*(6), 704-719.	Not older adults
51. Rochester, C. L. (2022). Does telemedicine promote physical activity? *Life (Basel, Switzerland)*, *12*(3), 425-.	Not a systematic review
52. Ruivo, J. A. (2014). Exergames and cardiac rehabilitation: A review. *Journal of Cardiopulmonary Rehabilitation and Prevention*, *34*(1), 2-20.	Not older adults
53. Sazlina, S.-G., Browning, C., & Yasin, S. (2013). Interventions to promote physical activity in older people with type 2 diabetes mellitus: a systematic review. *Frontiers in Public Health*, *1*, 71-71.	Included non-digital interventions
54. Schaffer, K., Panneerselvam, N., Loh, K. P., Herrmann, R., Kleckner, I. R., Dunne, R. F., Lin, P. J., Heckler, C. E., Gerbino, N., Bruckner, L. B., Storozynsky, E., Ky, B., Baran, A., Mohile, S. G., Mustian, K. M., & Fung, C. (2019). Systematic review of randomized controlled trials of exercise	No physical activity outcomes

	interventions using digital activity trackers in patients with cancer. *Journal of the National Comprehensive Cancer Network*, *17*(1), 57-63.	
55.	Tough, D., Robinson, J., Gowling, S., Raby, P., Dixon, J., & Harrison, S. L. (2018). The feasibility, acceptability and outcomes of exergaming among individuals with cancer: A systematic review. *BMC Cancer*, *18*(1), 1151–1151.	Not older adults
56.	Turan Kavradim, S., Özer, Z., & Boz, İ. (2020). Effectiveness of telehealth interventions as a part of secondary prevention in coronary artery disease: A systematic review and meta-analysis. *Scandinavian Journal of Caring Sciences*, *34*(3), 585-603.	Not older adults
57.	Vaes, A. W., Cheung, A., Atakhorrami, M., Groenen, M. T., Amft, O., Franssen, F. M., Wouters, E. F., & Spruit, M. A. (2013). Effect of 'activity monitor-based' counseling on physical activity and health-related outcomes in patients with chronic diseases: A systematic review and meta-analysis. *Annals of medicine*, *45*(5-6), 397-412.	Not older adults
58.	Valenzuela, T., Okubo, Y., Woodbury, A., Lord, S. R., & Delbaere, K. (2018). Adherence to technology-based exercise programs in older adults: A systematic review. *Journal of Geriatric Physical Therapy (2001)*, *41*(1), 49-61.	No physical activity outcomes
59.	Vargemidis, D., Gerling, K., Spiel, K., Abeele, V. V., & Geurts, L. (2020). Wearable physical activity tracking systems for older adults—A systematic review. *ACM Transactions on Computing for Healthcare*, *1*(4), 1-37.	No physical activity outcomes
60.	Vasquez, B. A., Betriana, F., Nemenzo, E., Inabangan, A. K., Tanioka, R., Garcia, L., Juntasopeepun, P., Tanioka, T., & Locsin, R. C. (2023). Effects of healthcare technologies on the promotion of physical activities in older persons: A systematic review. *Informatics for Health & Social Care*, *48*(2), 196-210.	No physical activity outcomes
61.	Vázquez, F. L., Otero, P., García-Casal, J. A., Blanco, V., Torres, Á. J., & Arrojo, M. (2018). Efficacy of video game-based interventions for active aging. A systematic literature review and meta-analysis. *PloS One*, *13*(12), e0208192–e0208192.	Not older adults
62.	Vázquez-de Sebastián, J., Ciudin, A., & Castellano-Tejedor, C. (2021). Analysis of effectiveness and psychological techniques implemented in mHealth solutions for middle-aged and elderly adults with Type 2 diabetes: A narrative review of the LIterature. *Journal of Clinical Medicine*, *10*(12), 2701-.	Not a systematic review
63.	Xu, L., Shi, H., Shen, M., Ni, Y., Zhang, X., Pang, Y., Yu, T., Lian, X., Yu, T., Yang, X., & Li, F. (2022). The effects of mHealth-based gamification interventions on participation in physical Activity: Systematic review. *JMIR mHealth and uHealth*, *10*(2), e27794-e27794.	Not older adults
