# Supplementary material for: The effectiveness of digital physical activity interventions in older adults: a systematic umbrella review and meta-meta-analysis
Source: Int J Behav Nutr Phys Act. 2024 Dec 18;21:144. doi: 10.1186/s12966-024-01694-4 (PMC11658456; doi:10.1186/s12966-024-01694-4)
Supplement: Supplementary file 3 — Additional file 3. Data extraction form. [file 12966_2024_1694_MOESM3_ESM.pdf]

Additional file 3: Data extraction form

General information

Title

Title of paper that data are extracted from

Lead author surname

Journal

Publication year

Country where review was conducted

Date search was conducted

Note: If not specified record end of date range for the search

Sources searched

Include databases, grey literature, citation searching etc

Search strategy (i.e. any limiters or restrictions)

e.g. date range, language?

What is the aim of the study?

AMSTAR2

AMSTAR2 Q1- Did the research questions and inclusion criteria for the review include the components of PICO?

1. Population
2. Intervention
3. Comparator group
4. Outcome

Describe the population

(e.g., age, gender, physical activity levels, chronic disease), include setting (e.g., workplace, healthcare)
Include any exclusions
Note: if only a subset of results meets inclusion criteria for our review answer for full paper as well as included subset.

Describe the intervention

(e.g., activity trackers, websites, mobile apps, SMS and exergame), include any non digital intervention components (telephone or face-to-face),
Include any exclusions
Note: if only a subset of results meets inclusion criteria for our review answer for full paper as well as included subset.

Describe the comparator group

i.e., inactive or active controls. If active what did they receive?
Include any exclusions

Describe the physical activity outcomes

(e.g., objective or self-reported minutes, frequency, intensity, guidelines, steps)
Include any exclusions

Describe any secondary outcomes

Secondary outcomes for our meta-review. Falls outcomes, (e.g., Berg Balance Scale, gait speed, sit to stand test), fitness outcomes (e.g., hand grip strength test, chair sit and reach test), sedentary behavior, sleep, nutrition, quality of life, mental health, cognition, physiological measures (e.g. heart rate, blood pressure)

List any other inclusion or exclusion criteria not included in PICO above

e.g., study designs

AMSTAR2 Q2 Did the report of the review contain an explicit statement that the review methods were established prior to the conduct of the review and did the report justify any significant deviations from the protocol?

For Partial Yes:
The authors state that they had a written
protocol or guide that included ALL the
following:
a) review question(s)
b) a search strategy
c) inclusion/exclusion criteria
d) a risk of bias assessment

For Yes:
As for partial yes, plus the protocol
should be registered and should also
have specified:
e) a meta-analysis/synthesis plan,
if appropriate, and
f) a plan for investigating causes
of heterogeneity (for example, subgroup analysis)
g) justification for any deviations
from the protocol

1. Yes
2. Partial yes
3. No

AMSTAR2 Q2 If no or partial yes please specify all components that are present, e.g., a), b), c) and g)

AMSTAR2 Q3 Did the review authors explain their selection of the study designs for inclusion in the review?

For Yes, the review should satisfy ONE of the following:
a) Explanation for including only RCTs
b) OR Explanation for including only NRSI (Non-randomised studies of interventions)
c) OR Explanation for including both RCTs and NRSI

1. Yes - option a)
2. Yes - option b)
3. Yes- option c)
4. No

AMSTAR Q4 Did the review authors use a comprehensive literature search strategy?

For Partial Yes (all the following):
a) searched at least 2 databases
(relevant to research question)
b) provided key word and/or

search strategy
c) justified publication restrictions
(e.g. language)

For Yes, should also have (all the following):
d) searched the reference lists / bibliographies of included studies
e) searched trial/study registries
f) included/consulted content experts in the field
g) where relevant, searched for grey literature
h) conducted search within 24 months of completion of the review

1. Yes
2. Partial yes
3. No

AMSTAR Q4 If no or partial yes please specify all components that are present, e.g., a), b), c) and g)

AMSTAR2 Q5 Did the review authors perform study selection in duplicate?

For Yes, either ONE of the following:
a) at least two reviewers independently agreed on selection of eligible studies and achieved consensus on which studies to include
b) OR two reviewers selected a sample of eligible studies and achieved good agreement (at least 80 percent), with the remainder selected by one reviewer.

1. Yes- option a)
2. Yes- option b)
3. No

AMSTAR2 Q6 6. Did the review authors perform data extraction in duplicate?

For Yes, either ONE of the following:
a) at least two reviewers achieved consensus on which data to extract from included studies
b) OR two reviewers extracted data from a sample of eligible studies and achieved good agreement (at least 80 percent), with the remainder extracted by one reviewer

1. Yes- option a)
2. Yes- option b)
3. No

AMSTAR2 Q7 Did the review authors provide a list of excluded studies and justify the exclusions?

For Partial Yes:
a) provided a list of all potentially relevant studies that were read in full-text form but excluded from the review

For Yes, must also have:

b) Justified the exclusion from
the review of each potentially
relevant study

1. Yes
2. Partial Yes
3. No

AMSTAR2 Q8 Did the review authors describe the included studies in adequate detail?

For Partial Yes (ALL the following):
a) described populations
b) described interventions
c) described comparators
d) described outcomes
e) described research designs

For Yes, should also have ALL the following:
f) described population in detail
g) described intervention in detail (including doses where relevant)
h) described comparator in detail (including doses where relevant)
i) described study's setting
j) timeframe for follow-up

1. Yes
2. Partial yes
3. No

AMSTAR2 Q8 If no or partial yes please specify all components that are present, e.g., a), b), c) and g)

Report the number of studies included in the review and the total participants across all studies

Total studies

Note: report both publications and unique studies if different
Note: if only a subset of results meets inclusion criteria for our review answer for included subset only.

Total enrolled participants

Note: if only a subset of results meets inclusion criteria for our review answer for included subset only.

List the included studies by first author surname and publication year e.g., Smith et al 2022

Note: if only a subset of results meets inclusion criteria for our review answer for included subset only.

What RoB tool was used?

AMSTAR2 Q9 Did the review authors use a satisfactory technique for assessing the risk of bias (RoB) in individual studies that were included in the review?

RCTs

For Partial Yes, must have assessed RoB
from
a) unconcealed allocation, and
b) lack of blinding of patients and
assessors when assessing
outcomes (unnecessary for
objective outcomes such as all cause mortality)

For Yes, must also have assessed RoB
from:
c) allocation sequence that was
not truly random, and
d) selection of the reported result
from among multiple
measurements or analyses of a
specified outcome

Note that Cochrane risk of bias and PEDro = YES

NRSI
For Partial Yes, must have assessed
RoB:
a) from confounding, and
b) from selection bias

For Yes, must also have assessed RoB:
c) methods used to ascertain
exposures and outcomes, and
d) selection of the reported result
from among multiple
measurements or analyses of a
specified outcome

Select all that apply (OR no)

1. Yes (RCT)
2. Partial yes (RCT)
3. Yes (NRSI)
4. Partial yes (NRSI)
5. No

AMSTAR2 Q10 Did the review authors report on the sources of funding for the studies included in the review?

For Yes
Must have reported on the sources of funding for individual studies included
in the review. Note: Reporting that the reviewers looked for this information
but it was not reported by study authors also qualifies

1. Yes
2. No

AMSTAR2 Q11 If meta-analysis was performed did the review authors use appropriate methods for statistical combination of results?

RCTs
For Yes:
a) The authors justified combining the data in a meta-analysis

b) AND they used an appropriate weighted technique (ok if they just state that weighting was used) to combine
study results and adjusted for heterogeneity if present (e.g., through random effects meta analysis).
c) AND investigated the causes of any heterogeneity (i.e., did they see if results differ by population, study and intervention characteristics).

For NRSI
For Yes:
a) The authors justified combining the data in a meta-analysis
b) AND they used an appropriate weighted technique to combine
study results, adjusting for heterogeneity if present
c) AND they statistically combined effect estimates from NRSI that
were adjusted for confounding, rather than combining raw data,
or justified combining raw data when adjusted effect estimates
were not available
d) AND they reported separate summary estimates for RCTs and
NRSI separately when both were included in the review

1. Yes
2. No
3. No meta-analysis conducted

AMSTAR2 Q11 For a no response, please specify which items are included e.g., a) and b) only

AMSTAR2 Q12 If meta-analysis was performed, did the review authors assess the potential impact of RoB in individual studies on the results of the meta-analysis or other evidence synthesis?

For Yes:
a) included only low risk of bias RCTs
b) OR, if the pooled estimate was based on RCTs and/or NRSI at variable
RoB, the authors performed analyses to investigate possible impact of
RoB on summary estimates of effect.

1. Yes - option a)
2. Yes - option b)
3. No
4. No meta- analysis conducted

Meta-analysis results

For each physical activity outcome (steps/day, mins/day, etc.) where a meta analysis was performed, report the below items:

Outcome & units: e.g. step count (steps/day)
Total studies: e.g. studies n=10
Total included participants: e.g. sample n=1200
Fixed vs random effects model: e.g. random effects
Effect size: e.g. SMD 0.19 (95% CI 0.1, 0.26), p=0.05, I squared= 0.00 (see below)

Example entry in textbox:
step count (steps/day), studies n=10, sample n=1200, random, SMD 0.19 (95% CI 0.22, 0.12), p=0.05, I=0.67

Further detail

Effect size

-Type, e.g. standardised mean difference (SMD), weighted mean difference (WMD)
-Overall effect size result (incl. units for WMD)
-95% CI = 95% confidence interval
-Test of heterogeneity (e.g. I squared or similar)

Outcome 2

Outcome 3

Outcome 4

Outcome 5

Outcome 6

Outcome 7

Outcome 8

Please report any additional analyses or outcomes of interest

e.g. Outcome 1 (steps/day): SMD was converted to WMD resulting in a difference of 1257 steps/day favouring the intervention group. this was equivalent to a 25% increase in baseline daily steps

For each secondary outcome (e.g., quality of life, sedentary behaviour mins/day) where a meta analysis was performed, report the below items:

Outcome & units: e.g. quality of life (score between 0-100)
Total studies: e.g. studies n=10
Total included participants: e.g. sample n=1200
Fixed vs random effects model: e.g. random effects
Effect size: e.g. SMD 0.19 (95% CI 0.1, 0.26), p=0.05, I squared= 0.00 (see below)

Example entry in textbox:
quality of life (score between 0-100), studies n=10, sample n=1200, random, SMD 0.19 (95% CI 0.22, 0.12), p=0.05, I=0.67

Further detail

Effect size
-Type, e.g. standardised mean difference (SMD), weighted mean difference (WMD)
-Overall effect size result (incl. units for WMD)
-95% CI = 95% confidence interval
-Test of heterogeneity (e.g. I squared or similar)

Outcome 2

Outcome 3

Outcome 4

Outcome 5

Outcome 6

Outcome 7

Outcome 8

Narrative results

Narrative synthesis of results only. Physical activity outcome measures

For each physical activity outcome (steps/day, mins/day, etc.) report the below items:
-outcome & units: e.g. step count (steps/day)

-Total studies: e.g. n=10
-Individual study results: e.g. favours intervention (FI) = 6. favours control (FC) = 0, non-significant (NS) = 4 (see below)

Example entry in textbox:
step count (steps/day), n=8, FI=7, FC=0, NS = 4, strong positive

Further explanation

Individual study results
For each outcome tally up totals of each type of result reported
-significant finding, favours intervention
-significant finding, favours control
-non-significant finding

Interpretation
An overall outcome result will need to be reported. This can be calculated using the below criteria:
- strong positive: >=75% of studies are significant, favours intervention
- positive: >50% of studies are significant, favours intervention
- weak positive: significant, favours intervention > significant favours control
- strong negative: >=75% studies are significant, favours control
- Negative >50% significant, favours control
- weak negative: significant favours control> significant, favours intervention

Outcome 2

Outcome 3

Outcome 4

Outcome 5

Outcome 6

Outcome 7

Outcome 8

Narrative synthesis of results only. Secondary outcome measures

For each secondary outcome e.g., quality of life report the below items:
-outcome & units: e.g. quality of life (score between 0-100)
-Total studies: e.g. n=10
-Individual study results: e.g. favours intervention (FI) = 6. favours control (FC) = 0, non-significant (NS) = 4 (see below)
-interpretation: e.g. positive findings

Example entry in textbox:
quality of life (score between 0-100), n=8, FI=7, FC=0, NS = 4, strong positive

Further explanation

Individual study results
For each outcome tally up totals of each type of result reported
-significant finding, favours intervention
-significant finding, favours control
-non-significant finding

Interpretation
An overall outcome result will need to be reported. This can be calculated using the below criteria:
- strong positive: >=75% of studies are significant, favours intervention
- positive: >50% of studies are significant, favours intervention
- weak positive: significant, favours intervention > significant favours control
- strong negative: >=75% studies are significant, favours control
- Negative >50% significant, favours control
- weak negative: significant favours control> significant, favours intervention

Outcome 2

Outcome 3

Outcome 4

Outcome 5

Outcome 6

Outcome 7

Outcome 8

Any adverse effects reported?

AMSTAR2 Q13 Did the review authors account for RoB in individual studies when interpreting/ discussing the results of the review?

For Yes:
a) included only low risk of bias RCTs
b) OR, if RCTs with moderate or high RoB, or NRSI were included the
review provided a discussion of the likely impact of RoB on the results

1. yes - option a)
2. yes - option b)
3. No

AMSTAR2 Q14 Did the review authors provide a satisfactory explanation for, and discussion of, any heterogeneity observed in the results of the review?

For Yes:
a) There was no significant heterogeneity in the results
b) OR if heterogeneity was present the authors performed an investigation of
sources of any heterogeneity in the results and discussed the impact of this
on the results of the review

1. yes- option a)
2. yes - option b)
3. No

AMSTAR2 Q15 If they performed quantitative synthesis did the review authors carry out an adequate investigation of publication bias (small study bias) and discuss its likely impact on the results of the review?

For Yes:
performed graphical or statistical tests for publication bias and discussed
the likelihood and magnitude of impact of publication bias

1. Yes
2. No
3. No meta-analysis conducted

AMSTAR2 Q16 Did the review authors report any potential sources of conflict of interest, including any funding they received for conducting the review?

For Yes:
a) The authors reported no competing interests OR
b) The authors described their funding sources and how they managed
potential conflicts of interest

1. yes- option a)
2. yes- option b)
3. No

Please record any additional notes or comments you have regarding this study (optional)
