# Supplementary material for: The effectiveness of digital physical activity interventions in older adults: a systematic umbrella review and meta-meta-analysis
Source: Int J Behav Nutr Phys Act. 2024 Dec 18;21:144. doi: 10.1186/s12966-024-01694-4 (PMC11658456; doi:10.1186/s12966-024-01694-4)
Supplement: Supplementary file 4 — Additional file 4. Table of review characteristics and results. [file 12966_2024_1694_MOESM4_ESM.pdf]

Additional File 4: Table of review characteristics and results

Study	Primary or secondary prevention	Technology category	Study designs	Measurement (Self-reported or device measured)	Control group	Number of studies (Total eligible)	Number of participants (Total eligible)	Meta analysis or narrative synthesis	Results	AMSTA R-2 rating
Aslam 2020	Both	m-health	RCT, quasi-experimental and pre-post	Both	Any	10	383	Narrative synthesis	Physical Activity, studies n=10, pos=8, neg=0, NS=2, strong evidence for a positive effect	Critically low
Baxter 2016	Primary	Any (sub analyses of e-health)	RCT, quasi-experimental or pre-post	Both	Any	103 (8)	Total *N* not reported (3962)	Narrative synthesis	Computer interventions: Self report, physical activity, studies n=5, pos= 3, neg= 0, NS= 2, moderate evidence for a positive effect<br><br>Pedometer/accelerometer interventions: Steps/day, studies n=3, pos= 3, neg= 0, NS= 0, strong evidence for a positive effect	Critically low
Buyl 2020	Primary	e-health	RCT or quasi-experimental	Both	Any	14 (6)	3679 (2682)	Narrative synthesis	Physical activity, studies n=6, pos=2, neg=0, NS=4, weak evidence for a positive effect	Low
Cooper 2018	Both	Wearable activity trackers	RCT	Both	Any	9 (8)	975 (888)	Meta-analysis	Accelerometers, total PA, studies n=4, participants, n=400, SMD= 0.43 (95% CI 0.19, 0.68), I^2=18.6%<br><br>Pedometers, total PA, studies n=4, sample	Critically low

									n=488, random, SMD 0.22 (95% CI -0.08, 0.51), I^2=48.2%	
D'Amore 2022	Both	e- or m-health	RCT	Both	Specific (face-to-face)	19 (17)	3405 (2524)	Meta-analysis	Steps/day, studies n=11, sample n=738, random, MD= 1440 (95% CI= 500, 2390), p=0.003, I=83%<br><br>total PA, studies n=8, sample n=2069, random, SMD= 0.17 (95% CI= 0.02, 0.32), p=0.03, I=46%<br><br>MVPA= studies n= 3, sample n= 475, random, SMD= 0.04 (95% CI= -0.14, 0.22), p=0.65, I=0%	Low
de Arenas-Arroyo 2021	Primary	e-health	RCT	Both	No intervention or other PA intervention	19 (18)	4937 (3120)	Meta-analysis	Steps/day, studies n = 8, participants n= 925: MD= 1616.28 (95% CI: 386, 2846), p=0.000, I^2 = 87.2%<br><br>MVPA (min/day), studies n = 5, participants n= 818, SMD = 0.49 (95% CI: 0.17, 0.80), p= 0.037, I^2= 57.8%.<br><br>MVPA (min/week), studies n= 5, participants n= 682, SMD= 0.31 (95% CI: 0.13, 0.48), p= 0.335, I^2 = 12.5%<br><br>total PA (min/week), studies, n=6,	Low

									participants n= 1834, SMD= 0.13 (95% CI: 0.01, 0.24), p= 0.055, I^2 = 51.3%	
Devi 2015	Secondary (CHD)	e- or m-health (internet-based)	RCT	Both	No interventio n	18 (8)	1392 (862)	Narrative synthesis	Physical activity, studies n=8, pos= 3, neg= 0, NS= 5, weak evidence for a positive effect	Critically low
Elavsky 2019	Both	m-health	RCT, Quasi-experimental or pre-post	Both	Any	52 (44)	5928 (4859)	Narrative synthesis	Healthy population, pre-post, physical activity, studies n=6, pos= 4, neg= 0, NS= 2, moderate evidence for a positive effect<br><br>Healthy population, RCT, physical activity, studies n=6, pos= 5, neg= 0, NS= 1, strong evidence for a positive effect<br><br>Special population, pre-post, physical activity, studies, n=12, pos= 6, neg= 1, NS= 5, weak evidence for a positive effect<br><br>Special population, RCT, physical activity, studies n=20, pos= 10, neg= 0, NS= 10, weak evidence for a positive effect<br><br>**All studies total,** Physical activity, studies n= 44, pos=25, neg=1, NS=18,	Critically low

									moderate evidence for a positive effect	
Heizmann 2023	Secondary (CVD)	Wearable activity trackers	RCT	Device measured	No intervention	16 (11)	1427 (884)	Meta-analysis	Steps/day, studies n = 11, participants n = 884. MD 1534 (95% CI 843, 2225), p<0,01, I^2=85%	Critically low
Hodkinson 2022	Secondary (cardio-metabolic conditions)	Wearable activity trackers	RCT	Device measured	No intervention	25 (9)	Total *N* not reported (1481)	Meta-analysis	Steps/day, studies n = 9, participants n = 1481, MD= 1656.14 (95% CI= 917, 2395), I^2=14.9%	Low
Kwan 2020	Both	e-health	RCT	Both	No intervention or less advanced e-health intervention	38 (23)	11194 (3812)	Meta-analysis	Steps/day, studies n= 11, participants n = 866, MD= 790, (95%CI = 300, 1280), p= 0.002, I^2= 12%<br><br>Subjective measures, total PA, studies n= 9, participants n = 2357, MD = 53.2 min/week, (95%CI= 30.18, 76.21), p< 0.00001, I^2= 25%<br><br>Objective measures, total PA, studies n= 5, participants n = 851, MD= 12.95 min/week, (95%CI = 10.09, 15.82), p< 0.00001, I^2= 0%	Critically low
Jonkman 2018	Primary	e- or m-health	RCT	Device measured	Any	12	1208	Narrative synthesis	Steps/day, studies n=11, pos= 7, neg= 0, NS = 4, moderate evidence for a positive effect<br><br>MVPA, studies n= 3, pos= 1, neg= 0, NS= 2, weak evidence for a negative effect	Critically low

									Light PA, studies n= 3, pos= 0, neg= 0, NS= 3, weak evidence for a positive effect<br><br>Moderate PA, studies n= 2, pos= 2, neg= 0, NS= 0, strong evidence for a positive effect<br><br>Vigorous PA, studies n= 2, pos =1, neg= 0, NS=1, weak evidence for a positive effect	
Larsen 2019	Both	Wearable activity trackers	RCT	Both	Specific-wearable with no feedback	21 (20)	2783 (2704)	Meta-analysis	Steps, studies n = 21, participants n = 2704, MD= 1297 (95% CI= 817, 1753), p <0.001, I^2= 0.792.<br><br>MVPA, studies n = 8, participants n = 1686, SMD= 0.34 (95% CI= 0.15, 0.52), p = 0.005, I^2 = 0.658	High
Liu 2020	Primary	Wearable activity trackers	RCT	Device measured	Any	10	Narrative synthesis: 1035<br><br>Meta-analysis: 259	Narrative synthesis and meta-analysis	Physical activity, studies n= 10, pos=9, neg=0, NS=1, strong evidence for a positive effect<br><br>Passive control, Steps/day, studies n= 2; participants n = 83, SMD= 1.23 (95%CI= 0.75, 1.70), p<0.001, I^2=0%<br><br>Active control, Steps/day, studies n= 4; participants n = 207, SMD= 1.27 (95%CI= -	Critically low

									0.51, 2.04), p=0.001, I^2=82%<br><br>MVPA, studies n= 3; participants n = 201, SMD= 0.22 (95%CI= -0.89, 0.10), p=0.61, I^2=89%	
Muellmann 2018	Primary	e- or m-health	RCT and quasi-experimental	Both	No intervention or other PA intervention	20	6671	Narrative synthesis	Physical activity, studies n=20, pos=16, neg=0, NS=4, strong evidence for a positive effect	Critically low
Oliveira 2020	Both	Wearable activity trackers	RCT	Device measured	No intervention or other PA intervention	23	2766	Meta-analysis	Steps/day, studies n= 23; participants n = 2766, MD= 1558 (95%CI= 1099, 2018), p<0.001, I^2=92%<br><br>[a]Secondary prevention, steps/day, studies n=14, SMD=0.54 (95%CI= 0.25, 0.82), p=0.001<br><br>[a]Primary prevention, steps/day, studies n=9, SMD=0.61 (95% CI= 0.25, 0.96, p=0.002<br><br>[a]3 months, steps/day, studies n= 4, SMD=0.14 (95% CI=- 0.08, 0.35), p=0.64, I^2=0%<br><br>[a]6 months, steps/day, studies n= 3, SMD=0.69 (95% CI= 0.16, 1.23), p=0.016, I^2=76%	Low

Patterson 2021	Secondary (CVD)	m-health (smart phone or tablet applications)	RCT and quasi-experimental	Both	Any	19 (8)	1543 (623)	Meta-analysis	MVPA, studies n = 6; participants n = 690, MD= 40.35 (95% CI= 1.03, 79.67), p = 0.04, I^2= 51%<br><br>Steps/day, studies n = 2, participants n = 67, MD= 2,390 (95% CI 1,006.9, 3,791.2), p = 0.0007, I^2= 0%	Critically low
Song 2018	Both	m-health	RCT and quasi-experimental	Both	Any	7	451	Narrative synthesis	Physical activity, studies n=5, pos=4, neg=0, NS=1, strong evidence for a positive effect<br><br>Steps, studies n= 3, pos=2, neg=0, NS=1, moderate evidence for a positive effect	Critically low
Stockwell 2019	Both	e- or m-health	RCT, quasi-experimental, and pre-post	Both	Any	22 (14)	1757 (577)	Meta-analysis	[a]RCT, total PA, studies n = 8; participants n = 450; SMD = 0.28 (95% CI 0.01, 0.56), p = 0.04, I2 = 47%<br><br>[a]Pre-post, total PA, studies n = 6 participants n = 159, SMD= 0.25 (95% CI= 0.09, 0.41), p = 0.002, I2 = 37%<br><br>[a]Follow up, RCT, total PA, studies n= 2, participants n = 255, SMD = 0.11 (95% CI=-0.14, 0.36), p = 0.39, I2 = 0%.	Low

									[a]RCT, steps/day, studies n= 6, participants, n= 383, MD= 401 (95% CI= −125, 926), p= 0.09, I2 = 0%<br><br>[a]Pre-post, steps/day, studies n= 2, n= 77, MD= 280 (95% CI= −508, 1068), p= 0.49, I2 = 0%<br><br>[a]RCT, MVPA, studies = 6, participants n = 694, SMD= 0.47 (95% CI= 0.32, 0.62), p < 0.001, I2 = 0%	
Tighe 2020	Secondary (non-communicable disease)	e- or m-health	RCT, quasi-experimental and pre-post	Both	Any	9 (4)	2058 (1631)	Narrative synthesis	Physical activity, studies n=4, pos=3, neg=0, NS=1, strong evidence for a positive effect	Critically low
Wu 2023	Primary	Wearable activity trackers	RCT	Device measured	No intervention	45 (43)	7144 (6264)	Meta-analysis	Steps/day, studies n=38, participants n= 5509, MD= 1430 (95% CI= 1092, 1767), I=85%, p<.001<br><br>total PA, studies n=4, participants n= 948, SMD= 0.21 (95% CI= 0.01 ,0.40), I=47%, p=0.04<br><br>MVPA, studies=18, participants n= 4193, SMD= 0.54 (95% CI=0.36, 0.72), I=85%, p<.001	Critically low

Yerrakalva 2019	Primary	m-health (smart phone or tablet applications)	RCT and quasi-experimental	Device measured	Any	6 (3)	486 (322)	Meta-analysis	Steps/day, studies n= 3, participants n=322, MD= 506 (95% CI= -80, 1092), I^2=80.5%,<br><br>[a]Steps/day follow up, studies n=2, participants n= 282, MD= 753 (95% CI -147, 1652), I^2=78%.	Critically low

Notes: [a]Additional meta-analysis outcomes that are not included in the meta-meta-analysis. Note: CVD= cardiovascular disease, CHD= coronary heart disease, RCT = randomised controlled trial, CI = confidence interval, SMD = standardised mean difference, MD = mean difference, MVPA = moderate-vigorous physical activity, total PA= total physical activity, pos = positive, neg = negative, n = number.
