# Supplementary material for: The effectiveness of digital physical activity interventions in older adults: a systematic umbrella review and meta-meta-analysis
Source: Int J Behav Nutr Phys Act. 2024 Dec 18;21:144. doi: 10.1186/s12966-024-01694-4 (PMC11658456; doi:10.1186/s12966-024-01694-4)
Supplement: Supplementary file 5 — Additional file 5. List of studies included in reviews and number of reviews they were included in. [file 12966_2024_1694_MOESM5_ESM.pdf]

Additional file 5: List of studies included in reviews and number of reviews they were included in

Study	Occurrences
Kim 2013	9
Lyons 2017	8
Bickmore 2013	7
Martin 2015	7
Wijsman 2013	7
Muller 2016	6
Cadmus-Bertram 2015	5
Suboc 2014	5
Tabak 2014	5
Croteau 2007	4
Dasgupta 2017	4
Demeyer 2017	4
Duscha 2018	4
Irvine 2013	4
King 2008	4
Knight 2014	4
Koizumi 2009	4
Maddison 2015	4
Peels 2013	4
Rowley 2017	4
Talbot 2003	4
Thompson 2014	4
Yates 2009	4
Frederix 2015	3
Kolt 2007	3
Lewis 2017	3
Mouton 2015	3
Mutrie 2012	3
Nolan 2017	3
Pinto 2005	3
Yates 2017	3
Croteau 2004	2
Cruz 2016	2
de Blok 2006	2
De Greef 2011	2
Fong 2016	2
Hornikx 2015	2
Houle 2012	2
Kawagoshi 2015	2
King 2007	2
King 2013	2

Kirk 2009	2
Kullgren 2014	2
McMurdo 2010	2
Mendelson 2014	2
Mendoza 2015	2
Nishiguchi 2015	2
Okamoto 2007	2
Roberts 2019	2
Rowley 2019	2
Strath 2011	2
Tew 2015	2
van der Weegen 2015	2
Van Dyck 2013	2
Van Dyck 2016	2
Van Stralen 2009	2
Warren 2014	2
Alonso-DomÃ¬nguez 2019	1
Andersen 2015	1
Antoine 2016	1
Antypas 2014	1
Araiza 2006	1
Armit 2005	1
Arsand 2010	1
Ashe 2015	1
Ball 2017	1
Barberan-Garcia 2014	1
Barnason 2009	1
Block 2015	1
Brickwood 2021	1
Broekhuizen 2016	1
Butler 2009	1
Chen 2017	1
Chow 2015	1
Christiansen 2020	1
Claes 2020	1
Cohen 2017	1
Cupples 2013	1
Danks 2016	1
Devi 2014	1
Donaldson 2014	1
Ellis 2019	1
Fayehun 2018	1
Finkelstein 2015	1
Finkelstein 2008	1
Fjeldsoe 2016	1
Freene 2013	1

Fukuoka 2015	1
Furber 2010	1
Gell 2017	1
Ginis 2016	1
Grau-Pellicer 2020	1
Grey 2019	1
Haeger 2017	1
Hageman 2005	1
Hansen 2000	1
Harris 2015	1
Holmen 2014	1
Homma 2016	1
Hong 2015	1
Hospes 2009	1
Izawa 2012	1
Jarvis 1997	1
Johnston 2016	1
Joosen 2018	1
Judice 2015	1
Kaminsky 2013	1
KanaÃ 2018	1
Karen 2014	1
Karlijn 2010	1
Katzmarzyk 2011	1
Kenfield 2019	1
Keogh 2014	1
King 2007	1
King 2020	1
King 2016	1
Kolt 2012	1
Kwan 2020	1
Lara 2016	1
Laubach 2009	1
Lear 2014	1
Lee 2007	1
Leskinen 2021	1
Li 2019	1
Lim 2016	1
Lindman 2021	1
Lindsay 2009	1
Liu 2021	1
Lynch 2019	1
Mansfield 2015	1
Martinson 2008	1
Maxwell-Smith 2018	1
McCoy 2017	1

McLellan 2018	1
Mendoza 2020	1
Muellmann 2019	1
Muntaner-Mas 2017	1
Nahm 2017	1
Nguyen 2009	1
Nicklas 2014	1
O'Brien 2015	1
Ozemek 2018	1
Parker 2016	1
Paul 2016	1
Paul 2017	1
Peels 2016	1
Pellegrini 2015	1
Pelssers 2013	1
Persell 2020	1
Poppe 2019	1
Reid 2012	1
Rotheram-Borus 2012	1
Rowley 2019	1
Ruiz 2012	1
Salvi 2018	1
Sawchuck 2008	1
Shake 2018	1
Shetty 2011	1
Skrepnik 2017	1
Slegers 2008	1
Southard 2003	1
Stuckey 2011	1
Sugden 2008	1
Tabak, opden Akker 2014	1
Tabak, Vollenbroek-Hutten 2014	1
Tess Harris 2017	1
Thomas 2017	1
Thomsen 2017	1
Tiedemann 2015	1
Uhm 2017	1
Van Dyck 2019	1
van Het 2014	1
Van Stralen 2011	1
Van Stralen 2010	1
Varas 2018	1
Vathsangam 2014	1
Verwey 2014	1
Vidoni 2016	1

Voncken-Brewater 2013	1
Vroege 2014	1
Weinstock 2011	1
Widmer 2017	1
Williams 2016	1
Yamada 2012	1
Zolfaghari 2012	1
Zutz 2007	1
