# Supplementary material for: The effectiveness of digital physical activity interventions in older adults: a systematic umbrella review and meta-meta-analysis
Source: Int J Behav Nutr Phys Act. 2024 Dec 18;21:144. doi: 10.1186/s12966-024-01694-4 (PMC11658456; doi:10.1186/s12966-024-01694-4)
Supplement: Supplementary file 6 — Additional file 6. AMSTAR-2 quality rating of included reviews. [file 12966_2024_1694_MOESM6_ESM.pdf]

Additional file 6: AMSTAR quality rating of included reviews

First author	AMSTAR 2 Q1 – PICO (population, intervention, comparator, outcome)	AMSTAR 2 Q2 – Protocol*	AMSTAR 2 Q3 – Study selection justification	AMSTAR 2 Q4 – Search strategy*	AMSTAR 2 Q5 – Study selection	AMSTAR 2 Q6 – Data extraction	AMSTAR 2 Q7 – Excluded studies*	AMSTAR 2 Q8 – Included studies	AMSTAR 2 Q9 – Risk of bias*	AMSTAR 2 Q10 – Funding sources	AMSTAR 2 Q11 – Meta-analysis methods*	AMSTAR 2 Q12 – Meta-analysis RoB	AMSTAR 2 Q13 – RoB interpretation*	AMSTAR 2 Q14 – Study heterogeneity	AMSTAR 2 Q15 – Publication bias*	AMSTAR 2 Q16 – Conflict of interest	Overall confidence rating
Aslam 2020	YYNY	N	N	PY	Y	N	N	Y	Y	N	N/A	N/A	N	N	N/A	Y	Critically low
Heizmann 2023	YYYY	Y	N	PY	Y	Y	N	Y	Y	N	Y	N	N	Y	Y	Y	Critically low
Wu 2023	YYYY	Y	N	N	N	Y	N	PY	Y	N	N	N	N	Y	Y	Y	Critically low
D'Amore 2022	YYYY	Y	N	PY	Y	Y	Y	PY	Y	N	Y	Y	Y	Y	N	Y	Low
Jonkman 2018	YYNY	N	N	N	N	N	N	PY	N	N	N/A	N/A	N	N	N/A	Y	Critically low
Yerrakalva 2019	NNNN	PY	N	PY	Y	Y	N	Y	Y	N	Y	N	Y	Y	N	Y	Critically low
Tighe 2020	YYYY	PY	Y	PY	Y	Y	N	PY	N	N	N/A	N/A	N	N	N/A	Y	Critically low
Stockwell 2019	YYYY	Y	N	PY	Y	Y	N	Y	Y	N	Y	Y	Y	Y	Y	N	Low
Song 2018	YYYY	N	N	PY	N	N	N	Y	N	N	N/A	N/A	N	N	N/A	N	Critically low
Patterson 2021	YYYY	Y	N	N	Y	Y	N	Y	Y (RCT and NRSI)	N	Y	N	N	Y	Y	Y	Critically low
Muellmann 2018	YYYY	Y	N	PY	Y	Y	N	Y	Y	N	N/A	N/A	N	N	N/A	Y	Critically low
Liu 2020	YYYY	PY	Y	PY	Y	Y	N	PY	Y	N	Y	N	Y	Y	N	Y	Critically low
Larsen 2019	YYYY	Y	Y	PY	Y	Y	Y	Y	Y	N	Y	Y	Y	Y	Y	Y	High
Kwan 2020	YYYY	N	N	PY	Y	Y	N	PY	Y	N	Y	Y	Y	Y	N	Y	Critically low
Oliveira 2020	YYYY	Y	N	PY	Y	Y	N	Y	Y	N	Y	Y	Y	Y	Y	Y	Low
Hodkinson 2022	YYYY	PY	N	PY	Y	Y	N	PY	Y	N	Y	Y	Y	Y	Y	Y	Low
Elavsky 2019	YYNY	N	N	PY	Y	N	N	Y	N	N	N/A	N/A	N	Y	N/A	Y	Critically low
Devi 2015	YYYY	N	N	N	Y	Y	Y	Y	Y	N	N/A	N/A	Y	Y	N/A	N	Critically low
de Arenas-Arroyo 2021	YYYY	Y	N	PY	Y	N	Y	PY	Y	N	Y	N	N	Y	Y	Y	Low
Cooper 2018	YYYY	N	Y	Y	Y	Y	N	PY	Y	N	Y	Y	N	Y	N	Y	Critically low
Buyl 2020	YYYY	Y	N	PY	Y	Y	Y	Y	Y	N	N/A	N/A	N	N	N/A	Y	Low
Baxter 2016	YYNY	Y	N	N	N	N	N	Y	Y (RCT and NRSI)	N	N/A	N/A	N	Y	N/A	Y	Critically low

Notes: * = critical domains, N = no, PY = partial yes, Y = yes, N/A = Not applicable
