# Supplementary material for: The effectiveness of digital physical activity interventions in older adults: a systematic umbrella review and meta-meta-analysis
Source: Int J Behav Nutr Phys Act. 2024 Dec 18;21:144. doi: 10.1186/s12966-024-01694-4 (PMC11658456; doi:10.1186/s12966-024-01694-4)

Additional File 7: Forest plots of meta-meta-analysis results for total PA, steps and MVPA

Table 1. Forest plot of meta-meta-review for total PA

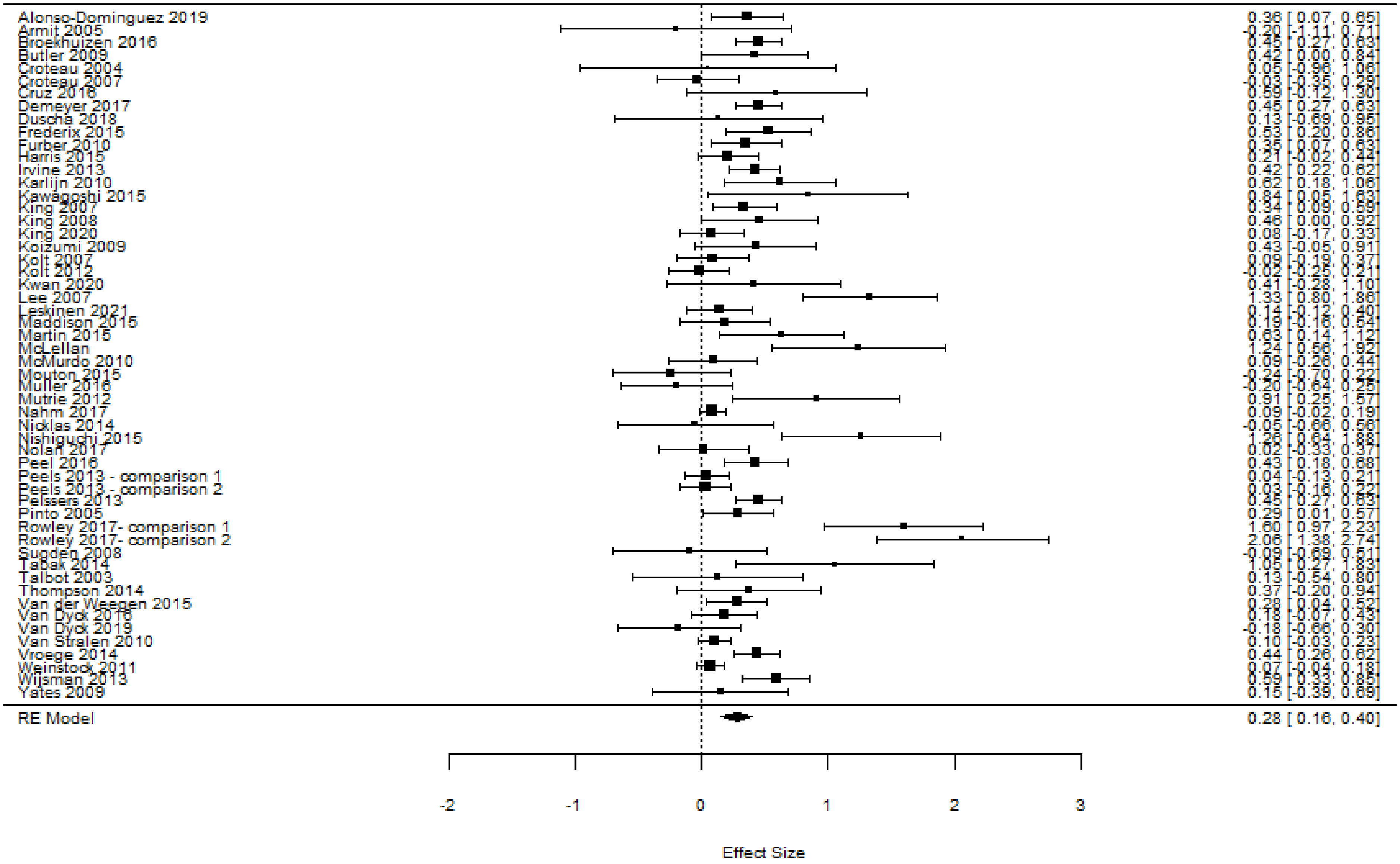

Table 2. Forest plot of meta-meta-review for steps

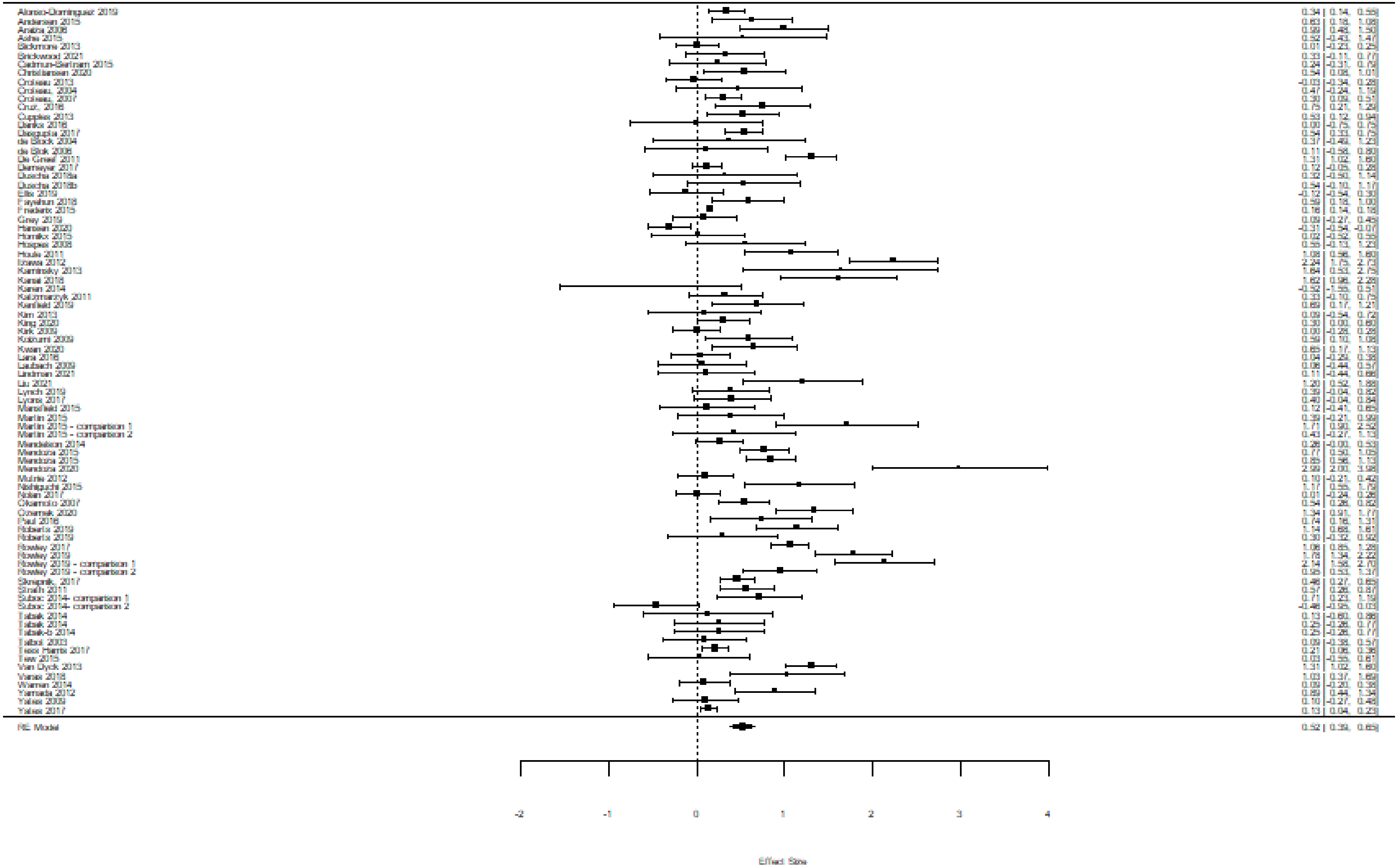

Table 3. Forest plot of meta-meta-review for MVPA

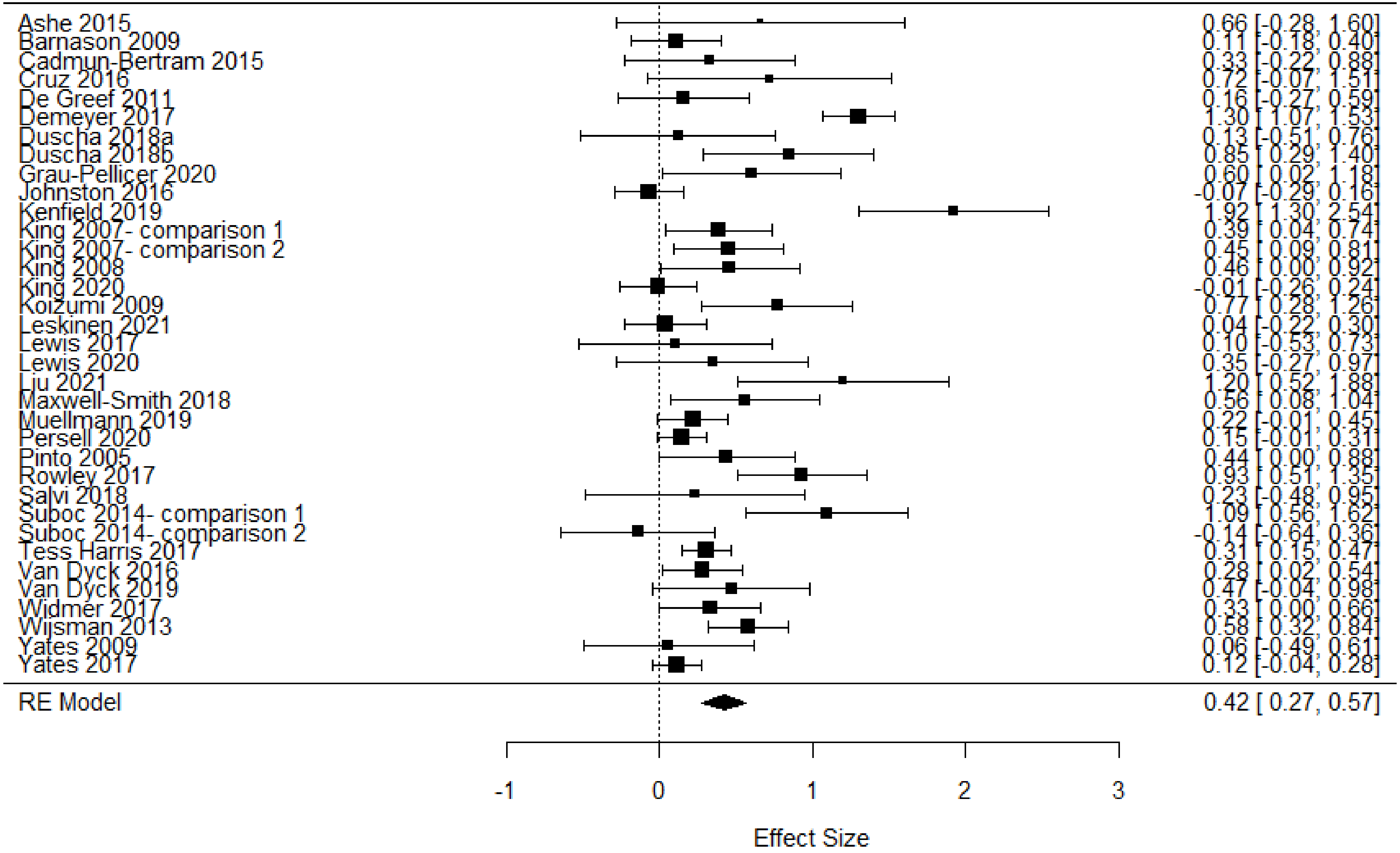

Supplement: Supplementary file 7 — Additional file 7. Forest plots of meta-meta-analysis results for total PA, steps and MVPA. [file 12966_2024_1694_MOESM7_ESM.pdf]
