# Supplementary material for: The effectiveness of digital physical activity interventions in older adults: a systematic umbrella review and meta-meta-analysis
Source: Int J Behav Nutr Phys Act. 2024 Dec 18;21:144. doi: 10.1186/s12966-024-01694-4 (PMC11658456; doi:10.1186/s12966-024-01694-4)

Additional File 8: Variance decomposition plots of meta-meta-analyses for steps, total PA and MVPA

Table 1.

Variance decomposition plot of the meta-meta-review for steps

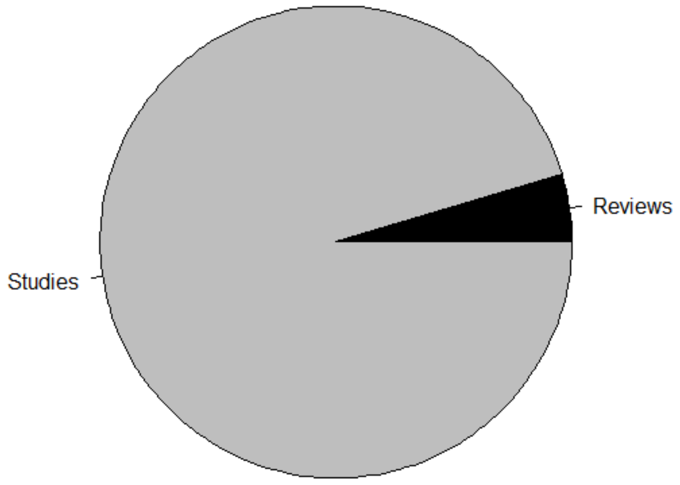

Table 2.

Variance decomposition plot of the meta-meta-review for total PA

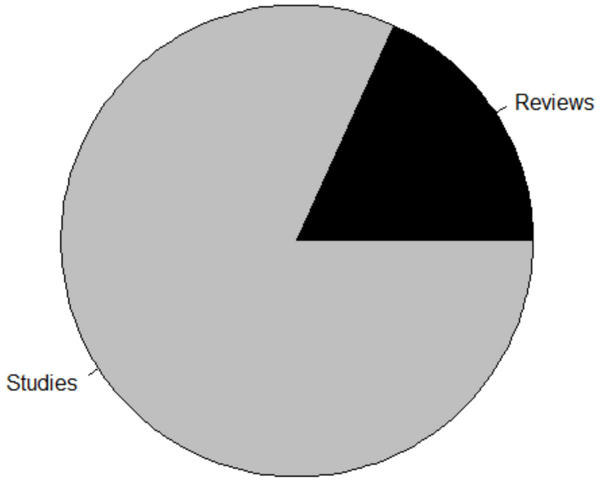

Table 3.

Variance decomposition plot of the meta-meta-review for MVPA

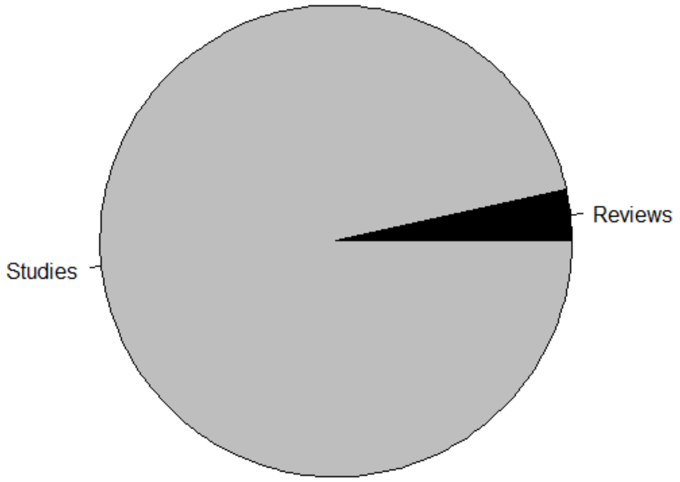

Supplement: Supplementary file 8 — Additional file 8. Variance decomposition plots of meta-meta-analyses for steps, MVPA and total PA. [file 12966_2024_1694_MOESM8_ESM.pdf]
